# Validity and reliability of subjective methods to assess sedentary behaviour in adults: a systematic review and meta-analysis

**DOI:** 10.1186/s12966-020-00972-1

**Published:** 2020-06-15

**Authors:** Esmée A. Bakker, Yvonne A. W. Hartman, Maria T. E. Hopman, Nicola D. Hopkins, Lee E. F. Graves, David W. Dunstan, Genevieve N. Healy, Thijs M. H. Eijsvogels, Dick H. J. Thijssen

**Affiliations:** 1 Radboud Institute for Health Sciences, Department of Physiology, Radboud University Medical Center, P.O. Box 9101, 6500 HB Nijmegen, The Netherlands; 2 Research Institute for Sport and Exercise Sciences, Liverpool John Moores University, Liverpool, UK; 3 Baker Heart & Diabetes Institute, Melbourne, Australia; 4 Mary MacKillop Institute for Health Research, Australian Catholic University, Melbourne, Australia; 5 The University of Queensland, School of Public Health, Brisbane, Australia

**Keywords:** Sedentary behaviour, Sitting, Measurement, Self-report, Reliability, Validity

## Abstract

**Background:**

Subjective measures of sedentary behaviour (SB) (i.e. questionnaires and diaries/logs) are widely implemented, and can be useful for capturing type and context of SBs. However, little is known about comparative validity and reliability. The aim of this systematic review and meta-analysis was to: 1) identify subjective methods to assess overall, domain- and behaviour-specific SB, and 2) examine the validity and reliability of these methods.

**Methods:**

The databases MEDLINE, EMBASE and SPORTDiscus were searched up to March 2020. Inclusion criteria were: 1) assessment of SB, 2) evaluation of subjective measurement tools, 3) being performed in healthy adults, 4) manuscript written in English, and 5) paper was peer-reviewed. Data of validity and/or reliability measurements was extracted from included studies and a meta-analysis using random effects was performed to assess the pooled correlation coefficients of the validity.

**Results:**

The systematic search resulted in 2423 hits. After excluding duplicates and screening on title and abstract, 82 studies were included with 75 self-reported measurement tools. There was wide variability in the measurement properties and quality of the studies. The criterion validity varied between poor-to-excellent (correlation coefficient [*R*] range − 0.01- 0.90) with logs/diaries (*R* = 0.63 [95%CI 0.48–0.78]) showing higher criterion validity compared to questionnaires (*R* = 0.35 [95%CI 0.32–0.39]). Furthermore, correlation coefficients of single- and multiple-item questionnaires were comparable (1-item *R* = 0.34; 2-to-9-items *R* = 0.35; ≥10-items *R* = 0.37). The reliability of SB measures was moderate-to-good, with the quality of these studies being mostly fair-to-good.

**Conclusion:**

Logs and diaries are recommended to validly and reliably assess self-reported SB. However, due to time and resources constraints, 1-item questionnaires may be preferred to subjectively assess SB in large-scale observations when showing similar validity and reliability compared to longer questionnaires.

**Registration number:**

CRD42018105994.

## Introduction

Regular physical activity reduces the risk of premature death, cardio- and cerebrovascular disease, metabolic disorders and some forms of cancer [1, 2]. Based on the overwhelming evidence, the World Health Organization recommend adults to perform ≥150-min moderate-intensity aerobic physical activity, or ≥ 75-min vigorous-intensity aerobic physical activity per week [3]. More recently, the importance of sedentary behaviour (SB) for health has emerged. High levels of SB are associated with an increased risk of premature death, cardiovascular disease, metabolic disorders and cancer [4–6], with especially strong associations in those who are physically inactive. These observations highlight the importance of accurately measuring physical activity *and* SB in order to understand their respective roles in health outcomes.

Various devices [7] and questionnaires [8] are available to assess physical activity. Since SB is a distinct behavioural entity and not simply reflective of the lack of sufficient physical activity, these measures may not directly assess SB [9]. Furthermore, in contrast with structured exercise, SB occurs habitually throughout the day, making valid assessment of SB challenging. SB is defined as any activity during awake time with an energy expenditure ≤1.5 METs (i.e. sitting or activities in reclining posture) [9, 10]. Patterns and total volume of SB can be assessed using objective measures such as thigh-worn accelerometers combining acceleration and posture, which is currently regarded as the gold standard to quantify free-living SB and to distinguish between sitting or lying, standing and physical activity [11]. Nonetheless, used in isolation, these objective measures do not distinguish between different domains (e.g. occupation, transportation and leisure time) and settings (e.g. TV viewing, car driving and sitting while reading) of SB. This is important since some settings of sitting, e.g. TV viewing and screen time, are more strongly associated with poor health outcomes compared to total sedentary time [12–14] and may serve as useful intervention targets. These observations emphasise the need for valid subjective measures to assess SB within the various domains and settings in which it occurs. Ideally, these measures should be taken in combination with objective assessments [15]. However, given this is not always possible or feasible, it is also important to understand the measurement metrics of self-report methods when they are used in isolation.

Several self-reported tools (i.e. questionnaires, logs and diaries) have been developed recently to measure SB. These tools vary from single-item questions to extensive questionnaires about SB considering various domains. Currently, some reviews compared the validity and reliability of these tools [15, 16]. However, previous reviews did not take the risk of bias across studies into account and did not combine the results into a meta-analysis. Knowledge about the validity, reliability and the quality of the studies performed is essential to plan, perform and correctly interpret results in this field of research, because measurement error may seriously impact study results. The aim of this systematic review and meta-analysis was to identify subjective methods to assess SB and, subsequently, to examine their validity and reliability to assess SB in adults. Where the sedentary time measured by subjective methods was compared to objective and other subjective methods. This overview will contribute to improved selection of appropriate subjective measures of SB (in relation to their research question), and to identify gaps of knowledge within this area of research.

## Methods

### Date source and literature search

A literature search was performed in databases of MEDLINE, EMBASE and SPORTDiscus. The search strategy combined three main search terms: sedentary behaviour, self-reported measures, and validity/reproducibility. The complete search strategy is shown in the Additional Table 1. The last search was performed on March 11th, 2020. All citations were imported into the bibliographic database of EndNote, version X7 (Thomas Reuters, New York City, NY). This review was registered in PROSPERO (number CRD42018105994) and the ‘Preferred Reporting Items for Systematic Reviews and Meta-Analyses’ (PRISMA) [98] guidelines were used to perform the systematic review and meta-analyses.

**Table 1:** Description of measurement tools to determine sedentary behaviour

Name of tool (reference)	Specific tool (no. of questions)^**a**^	Construct			Format^**c**^	Unit
		Domain^**b**^	Distinction in days (wk/wknd; work days)	Recall period		
***1-item questionnaires***						
**EEPAQ**; Elderly EXERNET Physical Activity Questionnaire [17]	Q (1)	To	yes	1 wk	T	Hrs (cat)
**GPAQ**: Global Physical Activity Questionnaire [18–24]	Q (1)	To	no	–	T	Hrs + min
**IPAQ** (short); International Physical Activity Questionnaire [25–27]	Q [1)	To	no	1 wk	T	Hrs + min
**Modified MOSPA-Q**; MONICA Optional Study on Physical Activity Questionnaire [28]	Q (1)	W	no	–	T	Hrs + min
**PPAQ**; Paffenbarger Physical Activity Questionnaire [29]	Q (1)	To	no	–	T	Hrs
**SED-GIH** [30]	Q (1)	To	no	–	T	Hrs (cat)
**SQ**; Single Question [31–33]	Q (1)	To	no	–	T	Hrs + min
**TASST**; TAxonomy of Self-report SB Tools [31, 34] 1) Single item total times; 2) Single item proportion; 3) TV time	Q (1: 1, 2: 1, 3: 1)	To	no	1 d 1 wk	T	Hrs + min %
**T-SQ**; Total sitting questionnaire [35]	Q (1)	To	no	7 d	T	Hrs + min
**TV-Q**; TV viewing [35]	Q (1)	To	no	–	T	Hrs + min
**YPAS**; Yale Physical Activity Survey for Older Adults [36]	I (1)	To	no	–	T	Hrs
**Clemes** et al. 2012 [33]	Q (1)	To	yes	–	T	Hrs + min
**Gao et al. 2017 (57)** 1) Single item proportion (3 months) 2) Single item proportion (1 day)	Q(1: 1, 2: 1)	W	no	1: 1 d 2: 3 mo	T	%
**Gupta** et al. 2017 [37]	Q (1)	To	no	3 d	T	Hrs + min
***2–9-item questionnaires***						
**AQuAA**; Activity Questionnaire for Adults and Adolescents [38]	Q (4)	L	no	1 wk	T	Hrs + min
**Cancer Prevention Study-3 Sedentary Time Survey** [39]	Q (4)	To + L	yes	1 yr	T	Hrs (cat)
**CHAMPS**; Community Health Activities Model Program for Seniors [36, 40]	Q (9)	L + other	no	4 wk	T	Hrs (cat)
**FPACQ**; Flemish Physical Activity Computerized Questionnaire [41, 42]	Q (3)	To + W + Tr	no	–	T	Hrs
**IPAQ** (long); International Physical Activity Questionnaire [26, 43–47]	Q (2)	To	no	1 wk	T	Hrs + min
**OPAQ**; Occupational Physical Activity Questionnaire [48]	Q (2)	W	no	–	T	Hrs
**OSPAQ**; Occupational Sitting and Physical Activity Questionnaire [28, 49–51]	Q (3)	W	no	1 wk	T	% of sitting
**PAS2**; Physical Activity Scale [52]	Q (2)	L + W	no	–	T	Hrs + min
**PASBAQ**; Physical Activity and Sedentary Behavior Assessment Questionnaire [53]	Q (3)	L + W	no	4 wk	T	Hrs + min
**PASB-Q**; Physical Activity and Sedentary Behavior Questionnaire [54]	Q (3)	L + W	no	–	T / Br	Hrs (cat) number
**PAST-U**; Past-day Adults’ Sedentary Time University [55]	Q (9)	L + Tr + W	no	1 d	T	Hrs + min
**PAT Survey**; Physical Activity and Transit Survey [56]	Q (2)	To	no	1 wk	T	Hrs + min
**RPAQ**; Recent Physical Activity Questionnaire [57, 58]	Q (4)	L + Tr	no	4 wk	T	Hrs (cat)
**Regicor Short Physical Activity Questionnaire** [59]	Q (4)	L	yes		T	Hrs
**SCCS PAQ**; Southern Community Cohort Study Physical Activity Questionnaire [60]	Q (6)	L + Tr + W	no	–	T	Hrs + min
**SITBRQ**: Workplace Sitting Breaks Questionnaire [61]	Q (2)	W	yes		Br	Freq + duration
**Stand Up For Your Health Questionnaire** [36, 62]	I (7)	L + Tr + W	no	1 wk	T	Hrs + min
**STAQ**; Sedentary, Transportation and Activity Questionnaire [63]	Q (7)	L + Tr + W	yes	4 wk	T	Hrs + min (cat)
**TASST**; TAxonomy of Self-report SB Tools [31] 4) Patterns; 5) Sum of domains	Q (4: 2, 5: 13)	H + L + Tr + W	no	1 d 1 wk	T / Bou	Hrs + min no. of bouts + duration
**Survey of older adults’ sedentary time** [64]	Q (8)	L + Tr + W	No	1 wk	T	Hrs + min
**Web-based physical activity questionnaire Active-Q** [65]	Q (8)	L + Tr + W	no	1 mo	T	Hrs + min (cat)
**WSWQ**; Percentage-Method Improves Properties of Workers’ Sitting- and Walking-Time Questionnaire [66]	Q (3/7)	L + W	yes	1 mo	T	Hrs + min %
**Clark** et al. 2011 [67]	Q (2)	W	yes	1 wk	T / Br	Hrs + min / freq
**Jefferis** et al. 2016 [68]	Q (4)	L + Tr	no	–	T	Hrs
**Lagersted**-**Olsen** et al. 2014 [69]	Q (4)	L + W	no	1 wk	T / Bou	Hrs + min
**Mielke et al. 2020** [70]	Q (5)	L + Tr + W	no	1 wk	T	Hrs + min
**Sudholx** et al. 2012 [71]	Q (2)	W	no	1 wk	T / Br	Hrs + min / freq
**Cartmel** et al. 1992 [72] Questionnaire A / B	Q (2)	Qa: H, L Qb: To	Qa: no Qb: yes	Qa: 1 yr Qb: -	T	Hrs + min
***≥10-item questionnaires***						
**ASBQ:** Adult Sedentary Behaviour Questionnaire [21]	Q (12)	L + Tr + W	yes	1 wk	T	Hrs + min
**D-SQ**; Domain-Specific Questionnaire [35]	Q (10)	L + Tr + W	yes	7 d	T	Hrs + min
**MPAQ**; Madras Physical Activity Questionnaire [73]	Q (19)	L + Tr + W	no	–	T	Hrs + min / freq
**MSTQ**; Multicontext Sitting Time Questionnaire [74]	Q (14)	L + Tr + W	no	–	T	Hrs + min
**PAFQ:** Physical Activity Frequency Questionnaire [75]	Q (140)	L + W	no	1 wk	T	Hrs + min
**PAST-WEEK-U** [76]	Q (63)	L + Tr + W	no	1 wk	T	Hrs + min
**NIGHTLY-WEEK-U** [76]	Q (63)	L + Tr + W	no	1 d	T	Hrs + min
**SBQ**; Sedentary Behaviour Questionnaire [24, 43, 77]	Q (18)	L + Tr + W	yes	–	T	Hrs + min (cat)
**SIT-Q**; Sedentary Behavior Questionnaire [78]	Q (20)	L + Tr + W	yes	1 yr	T / Br	Hrs + min (cat) / freq
**SIT-Q-7d**; last 7-d sedentary behavior questionnaire [79, 80]	Q (20)	L + Tr + W	yes	1 wk	T / Br	Hrs + min (cat) / freq
**STAR-Q** [81]	Q (17)	H + L + Tr + W	no	4 wk	T	Hrs + min
**TASST**; TAxonomy of Self-report SB Tools [31] [34] 6) Sum of behaviours	Q (13)	H + L + Tr + W	no	1 d 1 wk	T	Hrs + min
**WSQ**; Workforce Sitting Questionnaire [50, 82, 83]	Q (10)	L + Tr + W	yes	1 wk	T	Hrs + min
**Clark** et al. 2015 [84]	Q (10)	L + Tr + W	yes	1 wk	T	Hrs + min
**Clemes** et al. 2012 [33]	Q (10)	L + Tr + W	yes	–	T	Hrs + min
**Ishii** et al. 2018 [85]	Q (12)	L + Tr + W	Yes	1 wk	T	Hrs + min (cat)
**Marshall** et al. 2012 [86]	Q (10)	L + Tr + W	yes	–	T	Hrs + min
**Van Cauwenberg** et al. 2017 [87]	Q (12)	L + Tr	no	1 wk	T	Hrs + min
**Visser** et al. 2010 [88]	Q (20)	L + Tr + W	no	–	T	Hrs + min
***Logs and diaries***						
**7-day SLIPA Log**; (7-day Sedentary and Light Intensity Physical Activity Log) [89]	L	L + Tr + W	yes	1 d	T	Hrs + min
**BAR**; Bouchard Activity Record [90]	D	To	no	–	T	Hrs + min
**BeWell24 Self-Monitoring App** [91]	D	L + Tr + W	no	1 d	T	Hrs + min
**cpar24**; Computer-Based 24-Hour Physical Activity Recall Instrument [92]	D	L + Tr + W	no	1 d	T	Hrs + min
**EMA**; Ecological Momentary Assessment [93]	D	To	no	1 d	T	Hrs + min (cat)
**MARCA**; Multimedia Activity Recall for Children and Adults [32, 94]	I	L + Tr + W	no	1 d	T	Hrs + min
**PAMS**; Physical Activity Measurement Survey [95]	I	L + Tr + W	no	1 d	T	Hrs + min
**PDR**; Previous Day Recall [45]	I	L + Tr + W	no	1 d	T	Hrs + min
**Time Use Survey** [96]	D	L + Tr + W	no	1 d	T	Hrs + min
**Updated PDR**; Updated Previous Day Recall [97]	I	L + Tr + W	no	1 d	T	Hrs + min

^a^Q = questionnaire; L = log; D = diary; I = interview

^b^To = Total; H=Household; L = Leisure; Tr = Transport; W=Work

^c^T = Total time; Br = breaks; Bou = bouts

### Selection of papers

**Fig. 1:**
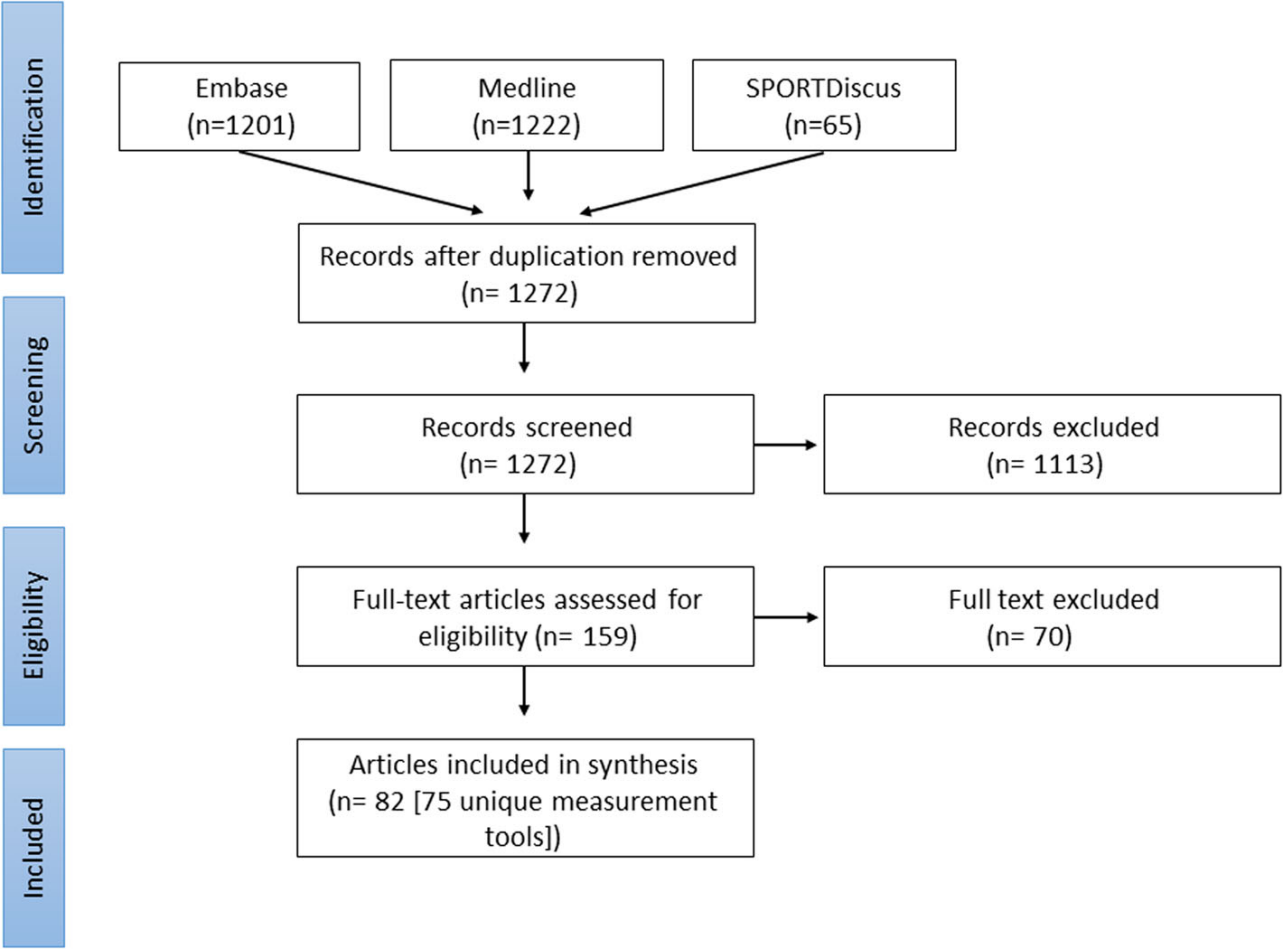
Flowchart of the inclusion of studies

After importing all citations in Endnote, duplicates were removed, and title, abstract and full text were independently screened by two reviewers (EB, YH). In case of disagreement, a third reviewer (TE) was consulted. Inclusion criteria were: 1) assessment of SB, 2) evaluation of subjective measurement tools, 3) being performed in healthy adults, 4) manuscript written in English, and 5) paper was peer-reviewed. Papers were excluded if the study did not aim to determine any construct of SB, when studies did not investigate the validation or reliability of the tool and/or the aim was to cross-cultural validate the subjective tool in different languages. A flowchart of the search strategy and the inclusion of manuscripts is presented in Fig. 1.

### Data extraction, synthesis and analysis

Study characters were extracted using an extraction form including: 1) study population, 2) number of participants, 3) gender and age, 4) the construct of SB measured (domain, setting, recall period, number of questions), 5) measurement outcomes (e.g. total sedentary time, breaks in sitting time, bouts), 6) comparison measure when validity was assessed, 7) interval between first and second measure when reliability was assessed, and 8) results of the measurement properties (e.g. intra correlation coefficients [ICC], correlations, mean bias with limits of agreement, kappa values and sensitivity/specificity). The extraction form was created by one (EB) and piloted by both reviewers (EB, YH). The pilot was performed using 10 randomly selected studies and changes were made to improve the extraction form. The quality of the studies was determined using the checklist with 4-point scale of COSMIN (Consensus-based Standards for the selection of health Measurement Instruments) criteria [99–101]. The COSMIN checklist contained items about the criterion validity (Additional Table 2) and reliability (Additional Table 3). For each item different design requirements and statistical methods were rated on quality using a 4-point scale. A methodological quality score per item was obtained by taking the lowest rating of any score per item (‘worse score counts’) [101].

**Table 2:** Construct validity of subjective sedentary behaviour measurement tools

Subjective tool	Study population		Validity			Quality of study
	N	gender male; mean age [SD] or age range; nationality	Comparison measure	Correlation (95%CI)	Other results	
***1-item questionnaires***						
**EEPAQ**; Elderly EXERNET Physical Activity Questionnaire [17] Lopez-Rodriguez et al. 2017	73	15%; 71.96 (5.48) yr; ESP	Actigraph GT1 M	0.574 P < 0.01		Poor
**GPAQ: Global Physical Activity Questionnaire** [21] Chu et al. 2018	78	31%; 20–65 yr; SGP	Actigraph GT3X-BL	Self-administered: 0.46 (0.18; 0.68) Interview administered: 0.12 (− 0.11; 0.33)	MD − 175.8 min (LoA − 556.1; 206.5) *More details are provided in study*	Poor
**GPAQ**: Global Physical Activity Questionnaire [20] Cleland et al. 2014	65	54%; 44 (14) yr; GBR	Actigraph GT3X	0.187 P = 0.135	MD − 348.7 min (LoA − 721.1; 23.7)	Poor
**GPAQ:** Global Physical Activity Questionnaire [24] Kastelic et al. 2019	42	88%; M: 38 (8) yr F: 50 (7) yr; SVN	ActivPAL 3	0.317 P = 0.041	MD − 165 min (LoA − 429; 99)	Fair
**GPAQ**: Global Physical Activity Questionnaire [18] Laeremens et al. 2017	122	45%; 35 (10) yr; BEL, ESP, GBR	Sensewear armband	*Mid-season*: 0.09 P > 0.05 *Summer:* 0.25 P < 0.01 *Winter:* 0.24 P < 0.01	MD 8 min (LoA −75; 92)	Poor
**GPAQ**: Global Physical Activity Questionnaire [22] Metcalf et al. 2018	108	31%; 49.4 yr (range: 19.8–68.7); USA	ActiGraph GT9X	0.19		Poor
**GPAQ**: Global Physical Activity Questionnaire [23] Rudolf et al. 2020	54	43%; 28.3 (12.2) yr; DEU	ActiGraph GT3X+	GPAQ with illustration of exemplary physical activities: 0.32 P = 0.02 GPAQ without illustration: 0.29 P = 0.03	GPAQ with illustration: − 9.3 min/day (LoA − 322.1; 303.5) GPAQ without illustration: − 18.3 min/day (LoA − 313.7; 277.1)	Poor
**GPAQ**: Global Physical Activity Questionnaire [19] Wanner et al. 2017	366	49%; 47.0 (15) yr; CHE	Actigraph GT3X+	0.47 P ≤ 0.001		Poor
**IPAQ** (short); International Physical Activity Questionnaire [26] Craig et al. 2003	2721	25–73%; 18–65 yr; 12 countries	Accelerometer (CSA model 7164)	Range: 0.07–0.61		Poor
**IPAQ** (short); International Physical Activity Questionnaire [43] Prince et al. 2018	313	0%; 42.8 (11.9) yr; CAN	ActiGraph GT3X	0.31 (P < 0.001)	*IPAQ*: MD 451.9–0.826* min	Poor
**IPAQ** (short); International Physical Activity Questionnaire) [44] Rosenberg et al. 2008	289	45%; 35.9 (11.3) yr; GBR, USA, NLD	Accelerometer (CSA model 7164)	0.34		Poor
**Modified MOSPA-Q**; MONICA Optional Study on Physical Activity Questionnaire [28] Chau et al. 2012	70	40%; 19–60+ yr; AUS	ActiGraph GTIM	0.52 P < 0.01		Poor
**PPAQ**; Paffenbarger Physical Activity Questionnaire [29] Simpson et al. 2015	419	49%; M: 43.8 (15.8) yr F: 44.3 (16.5) yr; USA	Actical	0.20 (0.14; 0.33)		Poor
**SED-GIH** [30] Larsson et al. 2018	284	33%; 42.9 (8.9) yr; SWE	ActivPAL	0.31 (95% CI 0.20–0.41), P < 0.001		Excellent
**SQ**; Single Question [32] Aguilar-Farias et al. 2015	37	34%; 74.5 (7.6) yr; AUS	ActivPAL 3	0.33; wk. 0.31; wknd 0.28		Fair
**SQ**; Single Question [33] Clemes et al. 2012	44	30%; 41.5 (12.8) yr; GBR	ActiGraph GT1M	wk: 0.70 (P < 0.001) wknd: 0.55 (P < 0.001)	ICC wk.: 0.82 (P < 0.001) wknd: 0.69 (P < 0.001)	Poor
**TASST**; TAxonomy of Self-report SB Tools [31] 1) Single item total times; 2) Single item proportion; 3) TV time Chastin et al. 2018	700	48%; 64–83 yr; GBR	ActivPal	*Previous day* 1) 0.20; 2) 0.28; 3) 0.24 *Previous wk* 1) 0.23; 2) 0.36; 3) 0.23 *Unanchored* 1) 0.20; 2) 0.32; 3) 0.26 All *P*-values< 0.001	*Previous day* 1) LoA − 147; 561 2) LoA − 22; 48 3) LoA 145; 733 *Previous wk* 1) LoA; − 150; 564 2) LoA; − 19; 43 3) LoA 81; 732 *Unanchored* 1) LoA − 135; 549 2) LoA − 19; 51 3) LoA 125; 705	Excellent
**T-SQ**; Total sitting questionnaire [35] Kozey-Keadle et al. 2012	13	75%; 46.5 (10.8) yr; USA	ActivPal	wk 0.41; wknd 0.55	Wk MD 40.5 min (− 125.2; 22.3) Wknd MD 147.4 min (− 228.3; − 66.6)	Poor
**TV-Q**; TV viewing [35] Kozey-Keadle et al. 2012	13	75%; 46.5 (10.8); USA	ActivPal	wk: 0.07; wknd: − 0.11		Poor
**YPAS**; Yale Physical Activity Survey for Older Adults [36] Gennuso et al. 2015	58	21% 75.1 (6.5) yr; USA	ActiGraph GT1M		kappa − 0.0003, (− 0.0025; 0.0019)	Poor
**Gao** et al. 2017 [102]	70	41.4%; 33.1 (10.7) yr; CHI, FIN	Thigh-mounted accelerometer	3 months: 0.53 (95% CI 0.34–0.68), P < 0.001 Previous day: 0.53 (95% CI 0.45–0.61) P < 0.001	3 months: MD 2.4% (LoA − 0.5%; 5.3%) Previous day: MD 2.2% (LoA 0.7%; 3.6%)	Poor
**Gupta** et al. 2017 [37]	183	60%; 44.9 (9.8) yr; DNK	Actigraph GT3X	0.32 P < 0.001	MD 204.1 min (LoA 112.4–0.6*min; 463.6+ 0.6*min)	Poor
***2–9-item questionnaires***						
**AQuAA**; Activity Questionnaire for Adults and Adolescents [38] Chinapaw et al. 2009	47	36%; 30.1 (3.6) yr; NLD	Actigraph model 7164		ICC 0.15	Poor
**Cancer Prevention Study-3 Sedentary Time Survey** [39] Rees-Punia et al. 2018	713	41%; 51.7 yr (range 31–72); USA	Actigraph GT3x accelerometer + diary	0.41 (0.35; 0.47)		Poor
**CHAMPS**; Community Health Activities Model Program for Seniors [40] Hekler et al. 2012	870	43%; 66–80+ yr; USA	Actigraph model 7164 and 71,256	0.12 P < 0.001	MD − 2841.6 min (LoA − 4476.7; − 1206.5)	Poor
**CHAMPS**; Community Health Activities Model Program for Seniors [36] Gennuso et al. 2017	58	21%; 75.1 (6.5) yr; USA	ActiGraph GT1M	0.14 P = 0.28	*CHAMPS:* MD −5.21 hrs (LoA − 2.2; − 8.3)	Poor
**FPACQ**; Flemish Physical Activity Computerized Questionnaire [41] Matton et al. 2007	81	77%; 22–78 yr; BEL	RT3 Triaxial Research Tracker + activity record	Employed / unemployed *Eat*: M 0.53 (P< 0.01); F 0.56 (P< 0.01) *Sleep* M 0.69 (P< 0.001); F 0.60 (P< 0.001) *Tv* M 0.69 (P< 0.001); F 0.83 (P< 0.001) *Retired* *Eat* M 0.33; F 0.15 *Sleep* M 0.57 (P< 0.01); F 0.51 (P< 0.05) *Tv* M 0.78 (P< 0.001); F 0.80 (P< 0.001)		Poor
**FPACQ**; Flemish Physical Activity Computerized Questionnaire [42] Scheers et al. 2012	405	41.4 (9.8) yr; BEL	SenseWear + electronic activity diary	*Total sedentary time* 0.54 (P < 0.001) *Screen time* 0.57 (P = 0.648) *Motorized transport* 0.58 (P < 0.001)		Poor
**IPAQ** (long); International Physical Activity Questionnaire [27] Chastin et al. 2014	69	67%; 41.1 (9.0) yr; GBR	ActivPal	0.159 (P = 0.193)	ICC 0.149	Good
**IPAQ** (long); International Physical Activity Questionnaire [82] Chau et al. 2011	95	37%; 18–60+ yr; AUS	Actigraph GT1M	Wk: 0.47 Wknd: 0.31 Total: 0.46		Poor
**IPAQ** (long); International Physical Activity Questionnaire [46] Cleland et al. 2018	228	57%; 71.8 (6.6) yr; GBR	Actigraph GT3X+	Wk: 0.70 (P < 0.01) Wknd: 0.26	Wk: MD −168.6 min (LoA − 451.8; 114.6) Wknd d: MD − 173.9 min (LoA − 441.6; 93.8)	Poor
**IPAQ** (long); International Physical Activity Questionnaire [26] Craig et al. 2003	2721	25–73%; 18–65 yr; 12 countries	Accelerometer (CSA model 7164)	Range: 0.14–0.51		Poor
**IPAQ** (long); International Physical Activity Questionnaire [44] Rosenberg et al. 2008	289	45%; 35.9 (11.3) yr; GBR, USA, NLD	Accelerometer (CSA model 7164)	0.33		Poor
**IPAQ** (long); International Physical Activity Questionnaire [47] Ryan et al. 2018	86	48%; 73.7 (6.3) yr; GBR	GENEA, (GENEactiv Original)	0.29	MD 27.6 (LoA ± 26.5 hrs/week)	Poor
**IPAQ** (long); International Physical Activity Questionnaire [25] Wanner et al. 2016	346	45%; 54.6 yr; CHE	Actigraph GT3X	0.42 P ≤ 0.001	MD 26.4 min (LoA − 12.0; 64.9)	Poor
**OPAQ**; Occupational Physical Activity Questionnaire [48] Reis et al. 2005	41	32%; 38.8 (9.9) yr; USA	Physical activity record	0.37		Poor
**OSPAQ**; Occupational Sitting and Physical Activity Questionnaire [28] Chau et al. 2012	76	40%; 19–60+ yr; AUS	ActiGraph GTIM	0.65 P < 0.01	MD 22 min (LoA − 141.53; 185.18)	Poor
**OSPAQ**; Occupational Sitting and Physical Activity Questionnaire [51] Jancey et al. 2014	41	41%; 18–50+ yr; AUS	ActiGraph GT3X + on the waist or thigh	0.58 (0.33; 0.75)	MD − 25.4 min (LoA − 784.7; 733.9)	Poor
**OSPAQ**; Occupational Sitting and Physical Activity Questionnaire [49] Pedersen et al. 2016	34	25%; 45.62 (10.96) yr; AUS	ActivPal	0.90	MD 3.16% (LoA − 21.4%; 15.1%)	Poor
**OSPAQ**; Occupational Sitting and Physical Activity Questionnaire [50] van Nassau et al. 2015	42	14%; 38 (11) yr; AUS	ActivPAL	Day 1: 0.37 (P < 0.05) Day 2: 0.48 (P < 0.05) Day 3: 0.35 (P < 0.05)	*only figures available for MD and LoA*	Fair
**PAS2**; Physical Activity Scale [52] Pedersen et al. 2017	330	38%; 46.7 (8.5) yr; DNK	Actiheart	0.197 (*P* = 0.053)	MD −2.3 hrs (LoA − 9.04; 4.34)	Poor
**PASBAQ**; Physical Activity and Sedentary Behaviour Assessment Questionnaire [53] Scholes et al. 2014	2175	46%; M: 52.7 (17.7) yr F: 51.8 (17.8) yr; GBR	ActiGraph GT1M	Sedentary time for different cut-off points *M* < 50 cpm 0.25 (0.19; 0.31); < 100 cpm 0.25 (0.19; 0.30); < 200 cpm 0.23 (0.17; 0.29) *F* < 50 cpm 0.31 (0.25; 0.37); < 100 cpm 0.30 (0.24; 0.35); < 200 cpm 0.27 (0.21; 0.32)		Poor
**PASB-Q**; Physical Activity and Sedentary Behavior Questionnaire [54] Fowles et al. 2017	32	19%; M: 63 (9) yr F: 55 (10) yr; USA	ActiGraph® GT3X	*Total SB:* 0.29 P = 0.13 *Breaks:* 0.02 P > 0.05		Poor
**PAST-U**; Past-day Adults’ Sedentary Time University) [55] Clark et al. 2016	57	53%; 26 (IQR 23; 31) yr; AUS	ActivPAL	0.63 (0.44; 0.76)	ICC 0.64 (0.45; 0.77) MD 0.08 hrs (LoA − 3.9; 4.1)	Good
**PAT Survey**; Physical Activity and Transit Survey [56] Yi et al. 2015	667	39%; 18–65+ yr; USA	ActiGraph GT3X	0.32 P < 0.001	MD: 49 min (LoA − 441; 343)	Poor
**RPAQ**; Recent Physical Activity Questionnaire [58] Besson at el. 2010	50	50%; F 34.3 (8.8) yr M 35.2 (9.9) yr; GBR	Doubly labeled water and accelerometerwith heart rate	0.27 (P = 0.06)	0.7 hrs (LoA 6 2.8)	Poor
**RPAQ**; Recent Physical Activity Questionnaire [57] Golubic et al. 2014	1923	30%; F: 54.0 (9.3) yr, M: 55.0 (9.9) yr; EUR	Actiheart	F: 0.20 (0.14; 0.25) M: 0.25 (0.19; 0.31)	F: MD −3.3 hrs (LoA − 9.0; 4.1) M: MD − 2.3 hrs (LoA − 8.3; 5.5) Total MD − 3.1 hrs (LoA − 9.6; 4.9)	Poor
**Regicor Short Physical Activity Questionnaire** [59] Molina et al. 2017	114	45%; 54.5 (12.1) yr; ESP	SenseWear Pro3 Armband	0.244 (P = 0.020)		Poor
**SCCS PAQ**; Southern Community Cohort Study Physical Activity Questionnaire [60] Buchowski et al. 2012	118	48%; 54.5 (8.4) yr; USA	RT3 Stayhealthy	Range 0.17–0.30		Poor
**SITBRQ**: Workplace Sitting Breaks Questionnaire [61] Pedisic et al. 2014	143	37%; 18–60+ yr; AUS	Actigraph GT1M	Freq: 0.24 (0.07; 0.40) Duration: 0.05 (− 0.12; 0.22)		Poor
**Stand Up For Your Health Questionnaire** [62] Gardiner et al. 2011	48	27%; 72.8 (8.1) yr; AUS	ActiGraph GTIM	0.30 (0.02; 0.54)	MD − 9.20 + 0.67 hrs (LoA ± 3.82)	Poor
**STAQ**; Sedentary, Transportation and Activity Questionnaire [63] Mensah et al. 2016	88	47%; 40.5 (14.3) yr; FRA	Actigraph GT3X+ + Log	*Total* 0.54 (P < 0.001) *Work* 0.88 (P < 0.001) *Transport* 0.35 (P = 0.001) *Leisure time* 0.19 (P = 0.09) *TV/DVD* 0.46 (P < 0.001) *Computer/tablet/video game* 0.42 (P < 0.001)	*Total* ICC 0.44 (0.25; 0.60)	Poor
**TASST**; TAxonomy of Self-report SB Tools [31] 4) Sum of domains; 5) Patterns Chastin et al. 2018	700	48%; 64–83 yr; GBR	ActivPal	*Previous day* 4) 0.23; 5) 0.17 *Previous wk* 4) 0.30; 5) 0.23 *Unanchored* 4) 0.16; 5) 0.02 All P-values< 0.001	*Previous day* 4) LoA − 273; 533 5) LoA − 472; 748 *Previous wk* 4) LoA − 413; 482 5) LoA − 529; 727 *Unanchored* 4) LoA-373; 529 5) LoA − 34; 980 All *P*-values < 0.001	Excellent
**Survey of older adults’ sedentary time** [64] Gennuso et al. 2016	44	36%; 70 (68–76) yr; USA	ActivPAL	0.06 (P = 0.72)	MD 0.31 hrs (LoA − 6.74; 7.37)	Fair
**Web-based physical activity questionnaire Active-Q** [65] Bonn et al. 2015	148	100%; 65.4 (8.7) yr; SWE	GENEA Accelerometer	0.19 (0.04; 0.34)	MD − 178 min (LoA − 606,25)	Poor
**WSWQ**; Percentage-Method Improves Properties of Workers’ Sitting- and Walking-Time Questionnaire [66] Matsoe et al. 2016	62	58%; F 35.8 (7.5) yr M 46.3 (8.0) yr; JPN	ActivPAL	*Time method* At work: 0.56 Work day, not at work: 0.51 Non-workday: 0.37 *Percentage method* At work: 0.65 Work day, not at work: 0.60 Non-workday: 0.53 All P < 0.05	*Time method* At work: MD −7 min (LoA − 241; 241) Non-workday: MD − 115 min (LoA − 588; 358) *Percentage method* At work: MD 35 min (LoA − 200; 269) Non-workday: MD − 56 min (LoA − 392; 281)	Good
**Cartmel** et al. 1992 [72] Questionnaire A Questionnaire B	24	38%; M 69 (66–80) yr; F 74 (59–83) yr; USA	Diary		Time difference Qa 230 min *P* < 0.001 Qb 40 min, *P* = 0.47	Poor
**Clark** et al. 2011 [67]	121	40%; Median 34.9 (28.5–46.0) yr; AUS	Accelerometer	*Sedentary time* 0.39 (0.22; 0.53) *Sedentary breaks* 0.26 (0.11; 0.44)	*Sedentary time* MD −2.75 + 0.47* hrs (LoA ±2.25)	Poor
**Ishii** et al. 2018 [85]	392	39.8%; 50.1 (7) yr; JPN	Active style Pro, HJA-350IT	*Total* 0.49, P < 0.001 *Workdays* 0.57, P < 0.001 *Non-workdays* 0.23, P < 0.001	*Total* MD − 13.4 min/d (LoA − 361.9; 335.2) *Workdays* MD − 0.4 min/d (LoA − 378.9; 378.1) *Non-workdays* MD − 49.2 min/d (LoA − 477.7; 379.2)	Poor
**Jefferis** et al. 2016 [68]	1377	100%; 79 (71–93) yr; GBR	ActiGraph GT3X +	0.17 P < 0.001	MD 300 min (LoA − 6; 607)	Poor
**Lagersted-Olsen** et al. 2014 [69]	26	53%; 40.9 (8.6) yr; DNK	ActiGraph GT3X+ + diary	*Work:* Total 0.081 (P = 0.699) Uninterrupted sitting 0.315(P = 0.126) *Leisure time at workday:* Total − 0.185 (P = 0.366) Uninterrupted sitting − 0.069 (P = 0.762) *Leisure day:* Total 0.100 (P = 0.626) Uninterrupted sitting 0.063 (P = 0.770)	*Work:* MD 0.0 (LoA −3.4; 3.4) *Leisure:* MD 2.4 (LoA − 7.8; 3.0) Uninterrupted sitting: MD 0.5 (LoA − 1.1; 2.1)	Poor
**Sudholz** et al. 2017 [71]	52	58%; 32.1 (9.9) yr; AUS	ActivPal	Sitting time 0.24 (− 1.0; 0.47) Breaks 0.39 (0.25; 0.74)		Good
***≥10-item questionnaires***						
**ASBQ;** Adult Sedentary Behaviour Questionnaire [21] Chu et al. 2018	78	31%; 20–65 yr; SGP	Actigraph GT3X-BL	Self-administered: 0.31 (− 0.02; 0.58) Interview administered: − 0.07 (− 0.37; 0.24)	MD 4.6 min/d (LoA − 431.2; 440.4) *More details see full study*	Poor
**D-SQ**; Domain-Specific Questionnaire [35] Kozey-Keadle et al. 2012	20	75%; 46.5 (10.8) yr; USA	ActivPal	wk 0.30; wknd:0.17	Wk 176 min (96.1; 256.9) Wknd 157.6 min (22.1; 293.0)	Poor
**MPAQ**; Madras Physical Activity Questionnaire [73] Anjana et al. 2015	520	53%; 44.4 (14.2) yr; IND	GT3X+ Triaxial	0.48 (0.32; 0.62)		Poor
**MSTQ**; Multicontext Sitting Time Questionnaire [74] Whitfield et al. 2013	25	44%; 34.5 (7.7) yr; USA	ActiGraph GT1M	Work 0.34 P = 0.13 Non-working 0.61 P= 0.01		Poor
**PAFQ:** Physical Activity Frequency Questionnaire [75] Verhoog et al. 2019	1752	49%; 60.5 (9.4) yr; CHE	GENEActive	Total minutes 0.37 (0.33; 0.41) Total % of time 0.39 (0.35; 0.43)	*No exact numbers available for MD and LoA, figure only*	Poor
**PAST-WEEK-U** [76] Moulin et al. 2019	25	12%; ≤ 19 yr: 64% 20–24 yr: 36%; CAN	ActivPAL4		MD 0.09 hrs/day (LoA − 5.38; 5.55)	Poor
**NIGHTLY-WEEK-U** [76] Moulin et al. 2019	23	4%; ≤ 19 yr: 48%; 20–24 yr: 52%; CAN	ActivPAL4		MD 0.21 hrs/day (LoA − 1.75; 2.17)	Poor
**SBQ:** Sedentary Behaviour Questionnaire [24] Kastelic et al. 2019	42	88%; M: 38 (8) yr F: 50 (7) yr; SVN	ActivPAL 3	0.018, P = 0.910	MD − 181 min (LoA − 467; 105)	Fair
**SBQ**; Sedentary Behaviour Questionnaire [43] Prince et al. 2018	313	5%; 42.8 (11.9) yr; CAN	ActiGraph GT3X	0.43 (P < 0.001)	MD 350.27–0.6685* min	Poor
**SBQ**; Sedentary Behaviour Questionnaire [77] Rosenberg et al. 2010	842	48%; M: 43.9 (8.0) yr F: 41.2 (8.7) yr; USA	Actigraph (model WAM 7164)	Wk − 0.02 (0.78) Wknd − 0.005 (0.93) Total − 0.01 (0.81)		Poor
**SIT-Q**; Sedentary Behavior Questionnaire [78] Lynch et al. 2014	34	41%; 38.0 (19.5) yr; CAN	1) Diary – postural definition 2) Diary MET-based definition	1) Postural definition *Total* 0.53 (P < 0.01) *Meals* 0.19 (P = 0.11) *Transportation* 0.37 (P < 0.01) *Work, study, and volunteering* 0.76 (P < 0.01) *Care* 0.49 (P < 0.01) *Leisure time* 0.26 (P = 0.03) 2) MET-based definition *Total* 0.52 (P < 0.01) *Meals* 0.29 (P = 0.01) *Transportation* 0.34 (P < 0.01) *Work, study, and volunteering* 0.75 (P < 0.01) *Care* 0.46 (P < 0.01) *Leisure time* 0.26 (P = 0.03)		Poor
**SIT-Q-7d**; last 7-d sedentary behavior questionnaire [79] Busschaert etl al. 2015	66	Adults: 36%; 47.7 (10.5) yr; Older adults: 61%; 72.2 (4.4) yr; BEL	ActivPAL	Adults: *Average day* 0.49 (0.18; 0.71) P = 0.004 *Wk* 0.52 (0.22; 0.73) P = 0.002 *Wknd* 0.36 (− 0.29; 0.40) P = 0.743 Older Adults: *Average day* 0.48 (0.16; 0.71) P = 0.005 *Wk* 0.50 (0.19; 0.72) P = 0.003 *Wknd* 0.38 (0.04; 0.64) P = 0.030		Fair
**SIT-Q-7d**; last 7-d sedentary behavior questionnaire [80] Wijndeale et al.2014	53	38%; 38.4 (11.3) yr; BEL	ActivPAL + domain log	Dutch version *Total* 0.52 P < 0.001 *Meals* 0.21 P > 0.05 *Transportation* 0.46 P < 0.001 *Occupation* 0.63 P < 0.001 *Screen time* 0.76 P < 0.001 *Other* 0.36 P < 0.05	Dutch MD 59 min (LoA − 4.81; 8.17)	Good
**STAR-Q** [81] Csizmadi et al. 2014	102	40%; M: 50.6 (6.9) yr F: 46.0 (8.6) yr; CAN	Doubly Labeled Water and 7-d activity diary	Total 0.40 *P* < 0.001 Occupational sitting 0.75		Poor
**TASST** TAxonomy of Self-report SB Tools [31] 6) Sum of behaviours Chastin et al. 2018	700	48%; 64–83 yr; GBR	ActivPal	*Previous day* 6) 0.23 *Previous wk* 6) 0.32 *Unanchored* 6) 0.33 All P-values< 0.001	*Previous day* 6) -651; 367 *Previous wk* 6) -755; 265 *Unanchored* 6) -725; 286	Excellent
**WSQ**; Workforce Sitting Questionnaire [82] Chau et al. 2011	95	37%; 18–60+ yr; AUS	Actigraph GT1M	Work: 0.45 workday: 0.34 non-work: 0.23 Total: 0.40	Total MD 44.55 (LoA − 295.31; 384.41) Work day MD 1.58 (LoA − 227.86; 231.02)	Poor
**WSQ**; Workforce Sitting Questionnaire [50] van Nassau et al. 2015	42	14%; 38 (11) yr; AUS	ActivPAL	Day 1: 0.25 (P > 0.05) Day 2: 0.29 (P > 0.05) Day 3: 0.30 (P > 0.05)	*No exact numbers available for MD and LoA, figure only*	Fair
**WSQ**; Workforce Sitting Questionnaire [83] Toledo et al. 2019	546	25%; 45.1 (16.4) yr; USA	ActivPAL		MD (95% CI): Work hours 47.9 min (39.2; 56.6) Non work hours on workdays − 38.3 (− 47.4; − 29.1) Non work hours on non-workdays − 106.7 (− 124.0; − 89.5) Kappa agreement (95% CI): 0.13 (0.08; 0.18)	Poor
**Clark** et al. 2015 [84]	700	45%; 59 yr (range 35–65+); AUS	ActivPAL3	0.46 (0.40; 0.52)	0.53*average hrs (LoA ±4,32 h)	Excellent
**Clemes** et al. 2012 [33]	44	30%; 41.5 (12.8) yr; GBR	ActiGraph	*Domain specific:* wk.: 0.54 (P < 0.001) wknd: 0.13 (P = 0.41)	ICC *Domain:* wk.: 0.64 (P < 0.001) wknd: 0.20 (P = 0.23)	Poor
**Marshall** et al. 2010 [86]	101	38%; F: 51–59 yr M: < 50 - > 60 yr; AUS	7-d behaviour log for correlation coefficient and ActiGraph GT1M for Bland-Altman plots	*Travel* F wk. 0.47; wknd 0.20 M wk. 0.64; wknd 0.15 *Work* F wk. 0.69; wknd 0.38 M wk. 0.74; wknd 0.13 *TV* F wk. 0.61; wknd 0.53 M wk. 0.50; wknd 0.33 *Computer* F wk. 0.74; wknd 0.64 M wk. 0.69; wknd 0.61 *Other Leisure* F wk. 0.26; wknd 0.42 M wk. 0.21; wknd 0.19	*F* Wk:-63.6 (− 395.5; 268.4) Wknd: 10.8 (− 396.0; 419.7)	Poor
**Van Cauwenberg** et al. 2014 [87]	442	45%; 74.2 (6.2) yr; BEL	Actigraph GT3X +	0.30 (P < 0.001)	MD − 81.88 (LoA − 364.16; 200.41) at 540 min/d	Poor
**Visser** et al. 2013 [88]	83	51%; 74.3 (6.9) yr; NLD	Actigraph Model GT3X	0.35 (*P* < 0.05)	MD − 2.1 hrs (LoA − 7.40; 3.25)	Poor
***Logs and diaries***						
**7-day SLIPA Log**; (7-day Sedentary and Light Intensity Physical Activity Log) [89] Barwais et al. 2014	22	48%; 26.5 (4.1) yr; USA	GT3X	0.86 (0.70; 0.94)	MD − 0.3 hrs (LoA − 2.1; 1.6)	Poor
**BAR**; Bouchard Activity Record [90] Hart et al. 2011	32	50%; F: 30.2 (9.5) yr M: 29.1 (7.9) yr; USA	ActivPAL	0.87 P < 0.05		Fair
**BeWell24 Self-Monitoring App** Toledo et al. 2017 [91]	17	85%; 49.0 (8.9) yr; USA	ActivPAL3c		ICC 0.35 (0.04; 0.56) MD − 160.4 min (LoA-179.8; − 141.0)	Poor
**cpar24**; Computer-Based 24-Hour Physical Activity Recall Instrument [92] Kohler et al. 2017	49	49%; 50 (22–69) yr; DEU	ActiGraph GT3X	0.54	MD − 31 min (LoA − 380; 319)	Poor
**EMA**; Ecological Momentary Assessment [93] Knell et al. 2017	168	33%; 43.4 (13.1) yr; USA	ActiGraph GT3X	0.16 (P = 0.03)		Poor
**MARCA**; Multimedia Activity Recall for Children and Adults [32] Aguilar-Farias et al. 2015	33	34%; 74.5 (7.6) yr; AUS	ActivPAL 3	day 0.63; wk.: 0.67; wknd: 0.47		Fair
**MARCA**; Multimedia Activity Recall for Children and Adults [94] Gomersall et al. 2015	58	52%; 28 (7.4) yr; AUS	ActivPAL	0.77 (0.64; 0.86) P < 0.01	MD 0.59 hrs (LoA − 2.35; 3.53)	Good
**PAMS**; Physical Activity Measurement Survey [95] Kim et al. 2017	1356	42%; 46.2 (SE 0.4) yr; USA	SenseWear Armband Mini	*Day 1:* 0.45 (P = 0.04) *Day 2:* 0.49 (P = 0.04)	LoA − 618.6; 176.0 min	Poor
**PDR**; Previous Day Recall [45] Kozey Keadle et al. 2014	15	47%; 33.1 (11.5) yr; USA	Direct observation		ICC *Total* 0.81 (0.58; 0.91) *Home* 0.96 (0.91; 0.98) *Work/School* 0.93 (0.86; 0.97 *Community* 0.71 (0.47; 0.86) *Household activity* 0.84 (0.69; 0.93) *Work* 0.88 (0.75; 0.94) *Education* 0.12 (− 0.29; 0.48) *Transportation* 0.62 (0.32; 0.81) *Leisure* 0.55 (0.23; 0.77)	Poor
**Time Use Survey** van der Ploeg et al. 2010 [96]	129	59%; 18–63 yr; AUS	ActiGraph GT1M	Household Day 1: 0.39 (P < 0.05) Day 2: 0.49 (P < 0.05) Leisure time Day 1: 0.56 (P < 0.05) Day 2: 0.47 (P < 0.05) Transportation Day 1: 0.50 (P < 0.05) Day 2: 0.42 (P < 0.05) Non-occupational sedentary time Day 1: 0.57 (P < 0.05) Day 2: 0.59 (P < 0.05)		Poor
**Updated PDR**; Updated Previous Day Recall [97] Matthews et al. 2013	88	46%; 41.3 (14.8) yr; USA	ActivPal	M 0.81 (0.05) F 0.81 (0.04)	M MD 0.72 hrs (LoA − 2.61; 4.05) F MD 0.75 hrs (LoA − 2.21; 3.71)	Good

F: female, M: male, MD: mean difference, LoA: limits of agreement

**Table 3:** Reliability of subjective sedentary behaviour measurement tools

First author (year) Measure examined	Reliability				Quality of study
	Interval	n	ICC (95%CI)	Other results	
***1-item questionnaires***					
**EEPAQ**; Elderly EXERNET Physical Activity Questionnaire [17] Lopez-Rodriguez et al. 2017	2 wk	73	0.68		Good
**GPAQ**; Global Physical Activity Questionnaire [21] Chu et al. 2018	1 wk	78	Self-administered: 0.68 (0.47; 0.82) Interview-administered: 0.78 (0.64; 0.88)		Good
**IPAQ** (short); International Physical Activity Questionnaire [26] Craig et al. 2003	8–10 d	2721		*Correlation* Range: 0.18–0.95	Fair
**IPAQ** (short); International Physical Activity Questionnaire) [44] Rosenberg et al. 2008	3–7 d	255/257	Wk 0.59 Wknd 0.72 Total 0.81		Good
**Modified MOSPA-Q**; MONICA Optional Study on Physical Activity Questionnaire [28] Chau et al. 2012	1 wk	75	0.54 (0.36; 0.68)		Good
**PPAQ**; Paffenbarger Physical Activity Questionnaire [29] Simpson et al. 2015	3–6 mo	130	0.71 (0.61; 0.74)	*Correlation* 3 mo 0.39 (0.33; 0.51) 6 mo 0.43 (0.43; 0.60)	Fair
**SED-GIH** [30] Larsson et al. 2018	5.2 d (min 1 d, max 16 d)	94	0.86 (95% CI 0.79–0.90)	*Weighted Kappa* 0.77 (95% CI 0.68–0.86)	Good
**SQ**; Single Question [32] Aguilar-Farias et al. 2015	1 wk	38	D 0.79; wk. 0.80; wknd: 0.78		Fair
**TASST**; TAxonomy of Self-report SB Tools [34] 1) Single item total times; 2) TV time Dontje et al. 2018	1d, 1 wk	18	Previous day recall: 1) 0.414 (0.227; 0.655) 2) 0.595 (0.412; 0.783) Previous week recall: 1) 0.531 (0.1; 0.794) 2) 0.856 (0.657; 0.944)		Poor
**YPAS**; Yale Physical Activity Survey for Older Adults [36] Gennuso et al. 2015	10 d	*58*	0.588 P < 0.001		Good
**Gao** et al. 2017 [102]	1 d	70		Day-to-day variation: 9.4% ± 11.4%	Poor
***2–9-item questionnaires***					
**AQuAA**; Activity Questionnaire for Adults and Adolescents [38] Chinapaw et al. 2009	2 wk	47	0.60 (0.40; 0.74)		Good
**CHAMPS**; Community Health Activities Model Program for Seniors [40] Hekler et al. 2012	6 mo	748	0.56		Fair
**CHAMPS**; Community Health Activities Model Program for Seniors [36] Gennuso et al. 2017	10 d	58	*CHAMPS:* 0.638 P < 0.001		Good
**FPACQ**; Flemish Physical Activity Computerized Questionnaire [41] Matton et al. 2007	2 wk	102	*Employed / unemployed* *Eat* M 0.74 (0.53; 0.86); F 0.67 (0.43; 0.82) *Sleep* M 0.84 (0.70; 0.92); F 0.83 (0.70; 0.91) *Tv* M 0.93 (0.86; 0.97); F 0.92 (0.84; 0.96) *Retired individuals* *Eat* M 0.24 (− 0.20; 0.61); F 0.14 (− 0.35; 0.58) *Sleep* M 0.94 (0.86; 0.98); F 0.90 (0.75; 0.97) *Tv* M 0.76 (0.49; 0.89); F 0.89 (0.72; 0.96)		Good
**IPAQ** (long); International Physical Activity Questionnaire [82] Chau et al. 2011	1 wk	95	Wk: 0.69 (0.56; 0.78) Wknd: 0.65 (0.51; 0.76) Total: 0.73 (0.61; 0.81)		Good
**IPAQ** (long); International Physical Activity Questionnaire [26] Craig et al. 2003	8–10 d	2721		Range: 0.28–0.93	Fair
**IPAQ** (long); International Physical Activity Questionnaire [44] Rosenberg et al. 2008	3–7 d	255/257	Wk 0.81 Wknd 0.84 Total 0.82		Good
**OPAQ**; Occupational Physical Activity Questionnaire [48] Reis et al. 2005	2 wk	41	0.78 (0.62; 0.87)		Fair
**OSPAQ**; Occupational Sitting and Physical Activity Questionnaire [28] Chau et al. 2012	1 wk	84	0.89 (0.83; 0.92)		Good
**OSPAQ**; Occupational Sitting and Physical Activity Questionnaire [51] Jancey et al. 2014	7 d	99	0.66 (0.49; 0.77)		Good
**OSPAQ**; Occupational Sitting and Physical Activity Questionnaire [49] Pedersen et al. 2016	1 wk	75	0.44 (0.24; 0.60)		Good
**PASB-Q**; Physical Activity and Sedentary Behavior Questionnaire [54] Fowles et al. 2017	7 d	35		*Correlation* Total SB: 0.85 Breaks: 0.86 Work: 0.88 Leisure: 0.66 All P < 0.05	Fair
**Regicor Short Physical Activity Questionnaire** [59] Molina et al. 2017	1 wk	114	0.908 (0.867; 0.937)		Fair
**RPAQ**; Recent Physical Activity Questionnaire [58] Besson at el. 2010	F 14.3 (3.7) d M 16.4 (5.9) d	131	0.76 (P < 0.001)	*No exact numbers available for MD and LoA, figure only*	Good
**SCCS PAQ**; Southern Community Cohort Study Physical Activity Questionnaire [60] Buchowski et al. 2012	12–15 mo	118		*Correlation* Total: 0.33, P = 0.002 In car/bus: 0.33, P = 0.002 At work: 0.48, P < 0.001 Viewing TV/movies: 0.53, P< 0.001 Using home computer: 0.25, P = 0.02 Other: 0.24, P = 0.02	Poor
**SITBRQ**: Workplace Sitting Breaks Questionnaire [61] Pedisic et al. 2014	7–14 d	96		*Correlation* Freq breaks: 0.71 (0.59; 0.79) Duration breaks: 0.59 (0.45; 0.71) *Cohen’s kappa* Freq breaks: 0.74 (0.64; 0.84) Duration breaks: 0.61 (0.38; 0.85)	Good
**Stand Up For Your Health Questionnaire** [62] Gardiner et al. 2011	7 d	48	Total: 0.52 (0.27; 0.70) TV viewing: 0.76 (0.62; 0.86) Computer use: 0.79 (0.65; 0.88) Reading: 0.74 (0.51; 0.86) Socializing: 0.38 (0.11; 0.60) Transport: 0.40 (0.14; 0.61) Hobbies: 0.35 (0.07; 0.58) Other: 0.04 (− 0.25; 0.32)		Fair
**STAQ**; Sedentary, Transportation and Activity Questionnaire [63] Mensah et al. 2016	1 mo	32	Total 0.52 (0.22; 0.73) Leisure 0.37 (0.03; 0.62) Transport 0.28 (− 0.06; 0.56) Work 0.71 (0.49; 0.84) *See article for more settings*		Good
**Survey of older adults’ sedentary time** [64] Gennuso et al. 2016	7 d	44	0.48 P < 0.001		Fair
**Web-based physical activity questionnaire Active-Q** [65] Bonn et al. 2015	3 wk	148	0.80 (0.74–0.86)		Good
**WSWQ**; Percentage-Method Improves Properties of Workers’ Sitting- and Walking-Time Questionnaire [66] Matsoe et al. 2016	1 wk	62	Non-working time: Time = 0.49 (0.28–0.66) Percentage = 0.71 (0.56–0.81) Non-working day: Time = 0.64 (0.47–0.76) Percentage = 0.78 (0.66–0.86)		Good
**Mielke** et al. 2020 [70]	7 d	78		Lin’s CCC 0.87 (0.81–0.92)	Poor
**Sudholz** et al. 2017 [71]	7 d	59	Sitting time 0.78 (0.65; 0.86) Breaks 0.65 (0.48; 0.78)		Good
***≥10-item questionnaires***					
**ASBQ**: Adult sedentary Behaviour Questionnaire [21] Chu et al. 2018	1 wk	84	*Self-administered:* Total 0.74 (0.51; 0.86) Work 0.70 (0.43; 0.84) Transport 0.59 (0.22; 0.78) Eating 0.73 (0.48; 0.86) TV 0.85 (0.73; 0.92) Computer 0.57 (0.32; 0.75) Other 0.33 (0.04; 0.57) *Interview-administered* Total 0.66 (0.37; 0.81) Work 0.89 (0.80; 0.94) Transport 0.78 (0.59; 0.88) Eating: 0.71 (0.47; 0.84) TV 0.81 (0.67; 0.89) Computer 0.62 (0.40; 0.78) Other 0.42 (0.13; 0.64)		Good
**MPAQ**; Madras Physical Activity Questionnaire [73] Anjana et al. 2015	1 mo	543	Total: 0.81 TV viewing: 0.67		Good
**MSTQ**; Multicontext Sitting Time Questionnaire [74] Whitfield et al. 2013	7.2 d (− 3; 13.9)	21	*Workday:* Total 0.76 (0.50; 0.89) Working/reading/studying: 0.83 (0.62; 0.93) TV/movie: 0.93 (0.84; 0.97) Computer/video games: 0.39 (0.00; 0.70) Transport: 0.97 (0.93; 0.99) Socializing: 0.27 (0.00; 0.62) *Non-working:* Total 0.72 (0.42; 0.87) Working/reading/studying: 0.65 (0.31; 0.84) Tv/movies: 0.85 (0.67; 0.94) Computer/video games: 0.84 (0.64; 0.93) Transport: 0.70 (0.40; 0.87) Socializing: 0.62 (0.27; 0.83)		Poor
**SBQ**; Sedentary Behaviour Questionnaire [77] Rosenberg et al. 2010	2 wk	49	*Weekdays:* Total: 0.85 (0.75; 0.91) TV: 0.86 (0.76; 0.92) Computer games: 0.83 (0.72; 0.90). Sit listen to music: 0.71 (0.54; 0.83). Sit and talk on telephone: 0.81 (0.68; 0.89). Work: 0.77 (0.63; 0.87). Reading: 0.64 (0.44; 0.78). Playing music: 0.90 (0.82; 0.94). Arts and crafts: 0.70 (0.53; 0.82). Sitting driving in car: 0.76 (0.61; 0.86). *Weekend* Total: 0.77 (0.63; 0.86) TV: 0.83 (0.72; 0.90). Computer games: 0.80 (0.67; 0.88). Sit listen to music: 0.67 (0.49; 0.80). Sit and talk on telephone: 0.73 (0.57; 0.84). Work: 0.64 (0.44; 0.61). Reading:0.48 (0.24; 0.67). Playing music: 0.93 (0.87; 0.96). Arts and crafts: 0.51 (0.27; 0.69). Sitting driving in car: 0.72 (0.56; 0.83).		Fair
**SIT-Q**; Sedentary Behavior Questionnaire [78] Lynch et al. 2014	1 mo	64	Total: 0.65 (0.49; 0.78) Meals: 0.60 (0.42; 0.74) Transportation: 0.59 (0.41; 0.73) Work, study, and volunteering: 0.86 (0.78; 0.91) Leisure: 0.61 (0.43; 0.74)		Good
**SIT-Q-7d**; last 7-d sedentary behavior questionnaire [79] Busschaert etl al. 2015	Adults: 14 ± 5 d Older adults: 9 ± 1 d	42	Adults: Range 0.06; 1.00 Older adults: Range − 0.20; 1.00		Poor
**SIT-Q-7d**; last 7-d sedentary behavior questionnaire [80] Wijndeale et al.2014	3.3 wk. (2; 8 wk)	Dutch: 53 English: 281	*Average day* *Dutch* Total: 0.68 (0.50; 0.81) Transportation: 0.58 (0.37; 0.74) Occupation: 0.66 (0.46; 0.79) Screen time: 0.50 (0.26; 0.68) Other leisure time: 0.52 (0.29; 0.70) Breaks occupation:0.26 (− 0.07; 0.54) Breaks TV viewing: 0.31 (− 0.01; 0.57) *English* Total: 0.53 (0.44; 0.62) Transportation:0.50 (0.40; 0.58) Occupation: 0.74 (0.67; 0.80) Screen time: 0.61 (0.53; 0.67) Other leisure time: 0.45 (0.35; 0.54 Breaks occupation: 0.12 (− 0.04; 0.28) Breaks TV viewing: 0.28 (0.15; 0.39) *See article for more settings and weekdays / weekend days*		Good
**STAR-Q** [81] Csizmadi et al. 2014	3 mo 6 mo	95 96	Total: 0.53 (0.37; 0.66) 0.45 (0.28; 0.59) Work: 0.69 (0.57; 0.78) 0.69 (0.57; 0.78) TV viewing: 0.72 (0.61; 0.80) 0.63 (0.49; 0.74) Computer: 0.60 (0.46; 0.71) 0.62 (0.48; 0.73) Reading: 0.56 (0.41; 0.68) 0.39 (0.21; 0.55)		Fair
**TASST** TAxonomy of Self-report SB Tools [34] Dontje et al. 2018	1 d, 1 wk	18	Previous day recall: Sum of behaviours 0.743 (0.591; 0.874) Previous week recall: Sum of behaviours 0.758 (0.462; 0.902)		Poor
**WSQ**; Workforce Sitting Questionnaire [82] Chau et al. 2011	1 wk	95	*Workday* Total: 0.65 (0.51; 0.75) Transport: 0.67 (0.54; 0.77) Work: 0.63 (0.49; 0.74) TV: 0.91 (0.87; 0.94) Computer: 0.56 (0.40; 0.69) Other leisure activities: 0.68 (0.55; 0.78) *Non-work* Total: 0.80 (0.72; 0.87) Transport: 0.60 (0.45; 0.72) Work: 0.50 (0.33; 0.64) TV: 0.79 (0.69; 0.85) Computer: 0.81 (0.73; 0.87) Other leisure activities: 0.59 (0.44; 0.71) *Total* 0.73 (0.61; 0.81)		Good
**Ishii** et al. 2018 [85]	2 wk	34	*Total* 0.74 (0.55–0.86) *Workday* Car 0.85 (0.71–0.92) Public transport 0.60 (0.33; 0.78) Work 0.89 (0.80; 0.95) TV 0.76 (0.58; 0.88) Computer 0.72 (0.51; 0.85) Leisure 0.45 (0.15; 0.68) Total 0.77 (0.60; 0.88) *Non-workday* Car 0.53 (0.24; 0.74) Public transport 0.20 (− 0.15; 0.78) Work − 0.07 (− 0.40; 0.28) TV 0.79 (0.63; 0.89) Computer 0.72 (0.51; 0.85) Leisure 0.46 (0.14; 0.69) Total 0.53 (0.24; 0.73)		Fair
**Marshall** et al. 2010 [86]	mean 11 d (range 7–28 d)	101	*Work* M 0.86 (0.79; 0.90) F 0.79 (0.73; 0.84) *TV* M wk. 0.65 (0.52; 0.75); wknd 0.62 (0.48; 0.73) *Computer* F wk. 0.63 (0.52; 0.71); wknd: 0.72 (0.64; 0.79) M wk. 0.62 (0.48; 0.73); wknd: 0.59 (0.44; 0.71)	Total sedentary time M Wk MD − 4.3 min (LoA − 189.2; 180.7) Wknd MD − 8.1 min (LoA − 195.0; 178.8) F Wk MD − 3.9 min (LoA − 235.4; 227.5) Wknd MD − 5.6 min (LoA − 125.1; 113.9)	Good
**Van Cauwenberg** et al. 2014 [87]	8 d (1.7)	428	Total 0.77 (0.57; 0.89) TV viewing: 0.92 (0.83; 0.96) Computer use: 0.76 (0.54; 0.88) Reading: 0.60 (0.29; 0.79) Hobbies: 0.57 (0.26; 0.78) Seated conversation/listening: 0.40 (0.04; 0.67) Telephone: 0.69 (0.43; 0.84) Public transport: 0.46 (0.11; 0.71) Driving car: 0.79 (0.59; 0.90) Passenger in car: 0.11 (− 0.27; 0.46) Household: 0.12 (− 0.18; 0.53) Resting: 0.20 (− 0.18; 0.53) Eating: 0.46 (0.11; 0.71)		Good
**Visser** et al. 2013 [88]	23 d (SD 8)	63	0.71 (0.57; 0.81)		Fair
***Logs and diaries***					
**BeWell24 Self-Monitoring App** [91] Toledo et al. 2017	2 wk	17	0.65 (0.43; 0.82)		Poor
**cpar24**; Computer-Based 24-Hour Physical Activity Recall Instrument [92] Kohler et al. 2017	3 hrs	67	0.75		Fair
**MARCA**; Multimedia Activity Recall for Children and Adults [32] Aguilar-Farias et al. 2015	0.5–1 hrs	38	2 days before 0.72; yesterday 0.96		Fair
**Time Use Survey** [96] van der Ploeg et al. 2014	7 d	134	Non-occupational 0.55 (0.42; 0.66) Occupational 0.63 (0.51; 0.72)		Excellent

F: female, M: male, MD: mean difference, LoA: limits of agreement, d: day, wk: week, mo: month

### Assessment of construct validity and reliability

Criterion validity was defined as the degree to which the outcome measure measures the construct it purposes to measure [103]. Thigh-worn accelerometry (e.g. activPAL) was considered as the gold standard for total sedentary time, as they can more accurately distinguish between sitting and standing [11]. Hip-, waist- and wrist-worn accelerometers are frequently used as criterion measure. However, these accelerometers are not sensitive enough to distinguish between stationary standing and sitting [104]. On these grounds, studies using only hip-, waist- and wrist-worn accelerometers as criterion measure were graded with a lower level of evidence. In addition, if validity results of both thigh-worn accelerometers or hip-, waist- and wrist-worn accelerometers were included in the study, only the results of the thigh-worn accelerometers were reported in this review.

Reliability was defined as the degree of consistency and reproducibility of a measurement tool. Test-retest reliability is often assessed using an ICC [103]. Since Pearson and Spearman correlation coefficients neglect systematic errors, the use of Pearson and Spearman correlation coefficient was considered as inadequate and these studies were graded with a lower level of evidence. In addition, if studies provided both ICCs and correlation coefficients, only ICCs were reported in this review. An ICC > 0.90 was considered as excellent, ICC between 0.75–0.90 was considered as good, ICC between 0.50–0.75 as moderate and > 0.50 as poor [105].

### Data analyses

A meta-analysis using random effects [106] was performed to assess the pooled validity of the 1-item questionnaires, 2 to 9-item questionnaires, ≥10-item questionnaires and logs/diaries. A random effect model was used because it was unlikely that included studies were functional equivalent and results of the included studies had a large heterogeneity. Only studies expressing validity as Pearson or Spearman correlation coefficients were included in this analysis. When no correlation coefficient was provided for total sedentary time, an (unweighted) mean was calculated based on correlation coefficients of all setting and domains. Finally, I^2^ was calculated, which describes the proportion of total variation in effect size that was due to systematic differences between effect sizes rather than by chance [106]. Stratified analyses including only studies examining questionnaires with a good-to-excellent quality were performed to investigate if the quality of the study affected the pooled validity. Meta-analyses were performed using R with ‘Meta-Analysis with Correlations’ (MAc) package, version 1.1.1.

## Results

### Search results

The literature search resulted in 2423 hits (Fig. 1). After excluding duplicates, 1272 studies were screened for title and abstract. Most papers were not eligible for this review because: *i.* the articles did not aim to determine SB, *ii.* no measurement properties were assessed, and/or *iii.* The study was performed in children or diseased populations. In total 82 studies and 75 self-reported measurement tools were included (Table 1).

### Attributes of the questionnaires, logs and diaries

The majority of the subjective measures were questionnaires and contained different domains and settings of SB (Table 2). Measurement tools differed regarding the timing (week vs weekend), recall period and number of questions. Nearly all self-reported measurement tools expressed SB in total sitting time (hrs/day or hrs/week). The PASB-Q, SITBRQ, SIT-Q, SIT-Q-7d, TASST and several other questionnaires [31, 54, 61, 67, 69, 71, 78, 79] included total sitting time, but also information about sitting bout duration or breaks in sitting time.

### Validity

**Fig. 2:**
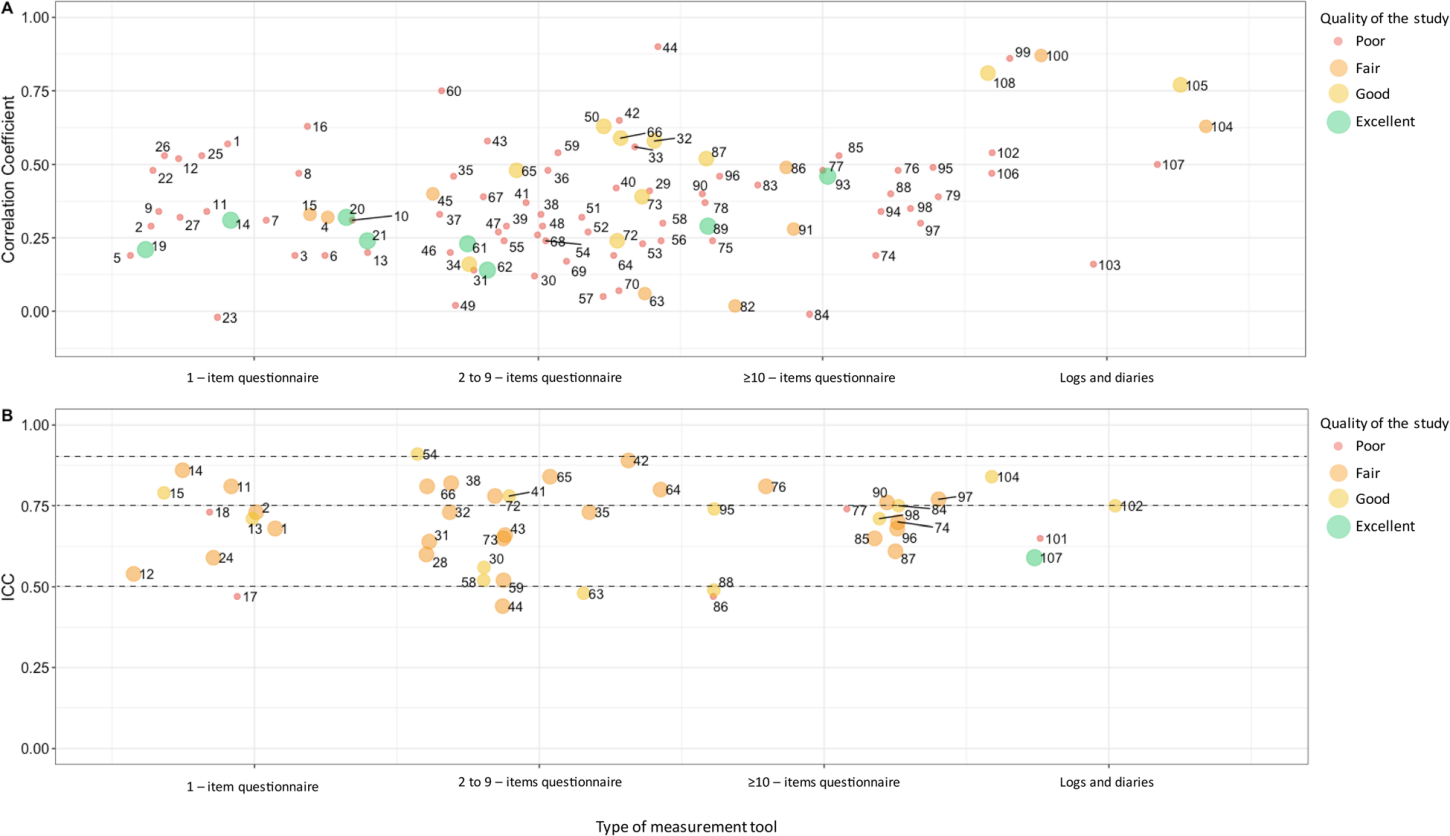
Overview of construct validity (**a**) and test retest reliability (**b**). 1 EEPAQ, Lopez-Rodriguez et al. 2017; 2 GPAQ, Chu et al. 2018; 3 GPAQ, Cleland et al. 2014; 4 GPAQ, Kastelic et al. 2019; 5 GPAQ, Laeremens et al. 2017; 6 GPAQ, Metcalf et al. 2018; 7 GPAQ, Rudolf et al. 2020; 8 GPAQ, Wanner et al. 2017; 9 IPAQ (short), Craig et al. 2003; 10 IPAQ (short), Prince et al. 2018; 11 IPAQ (short), Rosenberg et al. 2008; 12 Modified MOSPA-Q, Chau et al. 2012; 13 PPAQ, Simpson et al. 2015; 14 SED-GIH,; 15 SQ, Aguilar-Farias et al. 2015; 16 SQ, Clemes et al. 2012; 17 TASST Single item total times, Dontje et al. 2018; 18 TASST TV time, Dontje et al. 2019; 19 TASST Single item total times, Chastin et al. 2018; 20 TASST Single item proportion, Chastin et al. 2018; 21 TASST TV time, Chastin et al. 2018; 22 T-SQ, Kozey-Keadle et al. 2012; 23 TV-Q, Kozey-Keadle et al. 2012; 24 YPAS, Gennuso et al. 2015; 25 Single item proportion (3 months), Gao et al. 2017; 26 Single item proportion (1 day), Gao et al. 2017; 27 Gupta et al. 2017 [29]; 28 AQuAA, Chinpaw et al. 2009; 29 Cancer Prevention Study-3 Sedentary Time Survey, Rees-Punia et al. 2018; 30 CHAMPS, Hekler et al. 2012; 31 CHAMPS, Gennuso et al. 2017; 32 FPACQ, Matton et al. 2007; 33 FPACQ, Scheers et al. 2012; 34 IPAQ (long), Chastin et al. 2014; 35 IPAQ (long), Chau et al. 2011; 36 IPAQ (long), Cleland et al. 2018; 37 IPAQ (long), Craig et al. 2003; 38 IPAQ (long), Rosenberg et al. 2008; 39 IPAQ (long), Ruan et al. 2018; 40 IPAQ (long), Wanner et al. 2016; 41 OPAQ, Reis et al. 2005; 42 OSPAQ, Chau et al. 2012; 43 OSPAQ, Jancey et al. 2014; 44 OSPAQ, Pedersen et al. 2016; 45 OSPAQ, van Nassau et al. 2015; 46 PAS2, Pedersen et al. 2017; 47 PASBAQ, Scholes et al. 2014; 48 PASB-Q total SB, Fowles et al. 2017; 49 PASB-Q breaks, Fowles et al. 2017; 50 PAST-U, Clark et al. 2016; 51 PAT Survey, Yi et al. 2015; 52 RPAQ, Besson at el. 2010; 53 RPAQ, Golubic et al. 2014; 54 Regicor Short Physical Activity Questionnaire [47] Molina et al. 2017; 55 SCCS PAQ, Buchowski et al. 2012; 56 SITBRQ bout frequency, Pedisic et al. 2014; 57 SITBRQ bout duration, Pedisic et al. 2014; 58 Stand Up For Your Health Questionnaire, Gardiner et al. 2011; 59 STAQ, Mensah et al. 2016; 60 TASST, Sum of domains, Dontje et al. 2018; 61 TASST Sum of domains, Chastin et al. 2018; 62 TASST Patterns, Chastin et al. 2018; 63 Survey of older adults’ sedentary time, Gennuso et al. 2016; 64 Web-based physical activity questionnaire Active-Q, Bonn et al. 2015; 65 WSWQ Time method, Matsoe et al. 2016; 66 WSWQ Percentage method, Matsoe et al. 2016; 67 Sedentary time, Clark et al. 2011; 68 Sedentary breaks, Clark et al. 2011; 69 Jefferis et al. 2016; 70 Lagersted-Olsen et al. 2014; 71 Mielke et al. 2020; 72 Sitting time, Sudholz et al. 2017; 73 Sitting breaks, Sudholz et al. 2017; 74 ASBQ, Chu et al. 2018; 75 D-SQ, Kozey-Keadle et al. 2012; 76 MPAQ, Anjana et al. 2015; 77 MSTQ, Whitfield et al. 2013; 78 PAFQ sitting time, Verhoog et al. 2019; 79 PAFQ sitting proportion, Verhoog et al. 2019; 80 PAST-WEEK-U, Moulin et al. 2020; 81 NIGHTLY-WEEK-U, Moulin et al. 2020; 82 SBQ, Kastelic et al. 2019; 83 SBQ, Prince et al. 2018; 84 SBQ, Rosenberg et al. 2010; 85 SIT-Q, Lynch et al. 2014; 86 SIT-Q-7d, Busschaert et al. 2015; 87 SIT-Q-7d, Wijndeale et al.2014; 88 STAR-Q, Csizmadi et al. 2014; 89 TASST Chastin et al. 2018; 90 WSQ, Chau et al. 2011; 91 WSQ, van Nassau et al. 2015; 92 WSQ, Toledo et al. 2019; 93 Clark et al. 2015; 94 Clemes et al. 2012; 95 Ishii et al. 2018; 96 Marshall et al. 2010; 97 Van Cauwenberg et al. 2014; 98 Visser et al. 2013 [64]; 99 7-day SLIPA Log, Barwais et al. 2014; 100 BAR, Hart et al. 2011; 101 BeWell24 Self-Monitoring App, Toledo et al. 2017; 102 cpar24, Kohler et al. 2017; 103 EMA, Knell et al. 2017; 104 MARCA, Aguilar-Farias et al. 2015; 105 MARCA, Gomersall et al. 2015; 106 PAMS, Kim et al. 2017; 107 Time Use Survey, van der Ploeg et al. 2014; 108 Updated PDR, Matthews et al. 2013. The studies within each category are place randomly to avoid overlap when they are aligned. An ICC > 0.90 was considered as excellent, ICC between 0.75–0.90 was considered as good, ICC between 0.50–0.75 as moderate and > 0.50 as poor

A total of 80 studies examined the validity of one or more methods to assess SB, resulting in a comparison of 96 unique methods (Table 2). Of the 96 results, 5 were ranked with an excellent quality of the study, 7 studies with a good quality, 9 with a fair quality and 75 with a poor quality. The most important shortcoming of the validation studies was the use of an accelerometer (*n* = 62) to examine criterion validity of the method to assess SB. A total of 29 studies used the gold standard approach (thigh-worn accelerometer), three studies used diaries/logs and one used direct observation to assess construct validity. Most studies calculated correlation coefficients between the criterion measure and the self-reported questionnaire, which ranged between − 0.01 to 0.90 for total sedentary time and ranged between 0.02 to 0.39 for number of sedentary bouts or breaks (Table 3). Other studies used ICCs (*N* = 8), kappa values (*N* = 2), and sensitivity and specificity outcomes (*N* = 1) to determine the validity, and some added Bland-Altman plots with a mean difference and limits of agreement to examine the accuracy of the method to assess SB (*N* = 48). Figure 2a provides an overview of the correlation coefficient of all individual studies combined with the quality of the study.

### Meta-analyses

The correlation coefficients of logs and diaries (correlation coefficient estimate [*R*] = 0.63 [95% CI 0.48–0.78], I^2^: 95%) were substantially higher than the coefficients of the questionnaires (*R* = 0.35 [95% CI 0.32–0.39], I^2^: 90%). Furthermore, correlation coefficient estimates of the questionnaires with ≥10-item (*R* = 0.37 [95% CI 0.30–0.43], I^2^: 86%)) did not differ much from the questionnaires with fewer items (1-item questionnaire *R* = 0.34 [95% CI 0.30–0.39], I^2^: 68%; 2 to 9-item questionnaires *R* = 0.35 [95% CI 0.29–0.41], I^2^: 93%) (Fig. 2a). Stratified analyses, including only studies examining questionnaires with a good-to-excellent quality, revealed similar results (*R* questionnaires = 0.35 [95% CI 0.28–0.42], I^2^: 87%).

### Reliability

Reliability for total sitting time and number of breaks in sitting time was determined in 44 studies. One study was rated with excellent quality; other studies were rated with good (*n* = 27), fair (*n* = 16), and poor (*n* = 8) quality. Most studies with a lower quality of the study were limited by a small sample size and calculation of correlation coefficients instead of ICCs. The time interval between the first and second assessment ranged between 0.5 h and 15 months, but most studies had an interval of 1–2 weeks (*n* = 40, Table 3). The majority of the studies calculated the ICC to examine the test-retest reliability of SB, but some studies used correlation coefficients (*N* = 6), Bland-Altman plots with mean difference and limits of agreement (*N* = 2), and kappa values (N = 2). The ICC of the test-retest reliability of the subjective measures of SB ranged between 0.44 and 0.91 (Table 3, Fig. 2b). The ICC estimates were comparable between the logs and diaries, ≥10-items questionnaires, 2 to 9-item questionnaires, and 1-item questionnaires.

## Discussion

Time spent in SB has markedly increased over the last few decades and is expected to continue to increase even further [107]. Since SB is associated with many adverse health outcomes [4–6], exposure to excessive levels of SB represents an emerging health threat, particularly in the least physically active [108]. To improve quality and guide future studies in this rapidly expanding area of research, this systematic review assessed the validity and reliability of subjective measures of SB, taking the methodological quality into account. We present the following observations. First, despite the presence of several measures to assess SB, significant variability in measurement properties and quality of the studies is present. Second, criterion validity of the subjective measures ranged between poor to excellent (*R* range − 0.01 to 0.90), in which the quality of most studies (i.e. level of evidence) was poor. Third, the validity of the logs/diaries was more favourable compared to the validity of questionnaires, with little improvement in validity of questionnaires when including multiple questions. Fourth, a moderate-to-good reliability was found for questionnaires and logs/diaries, with the quality of these studies being largely fair-to-good. Taken together, logs and diaries are recommended to validly and reliably assess SB when only self-report measures are available. However, considering limitations pertaining to logs and diaries (e.g. time constraint, resources), one may prefer using questionnaires in larger scaled observations.

### Validity of measures of SB

This meta-analysis showed that the overall validity for instruments to assess SB characteristics was moderate to low. These observations raise the question whether these results relate to the poor validity of methods to assess SB per se or the poor quality of the studies that were included. Excluding studies with lower quality from our meta-analyses reinforced the poor-to-moderate validity of the various methods, suggesting measures of SB possess poor validity. It is important to indicate that questionnaires examining physical activity show similarly poor level of validity [8]. This highlights the difficulty of examining subjective physical (in) activity behaviours with questionnaires, a finding that seems present across the whole physical activity spectrum: from SB to exercise. Due to the low validity and the large variation in quality, the results of different studies are difficult to compare or harmonise. More importantly, the large variety in validity and questionnaire characteristics (i.e. type and context of SBs) prevents the identification of one (or few) questionnaire(s) that can be recommended for all type of future research that aim to examine SB.

Factors explaining the poorer variation in validity of the questionnaires versus diaries/logs may relate to differences in qualitative attributes (e.g. recall period and questions/formats). For example, diaries/logs typically adopt a short recall period (e.g. every 15–30 min), whilst questionnaires are often filled in covering a longer recall period (i.e. day, week, and/or month). Consequently, diaries and logs are less reliant on long-term recall and can more accurately capture sporadic and intermittent behaviours. This fits with the higher validity of diaries/logs versus questionnaires. Unfortunately, this approach of using diaries/logs comes with the cost of high participant burden (in time), which subsequently may limit the response and compliance rate and introduce reporting bias. Another potential limitation of logs/diaries is that repeatedly filling in SBs may influence participants’ behaviour and cause (unwanted) adjustment of SB. These factors should be considered when deciding on the preferred way to assess SB in a future study.

Previous work-related poor validity of questionnaires to systematic and random error, specifically reporting and recall bias which may lead to a low agreement with over- and underestimation (Table 2). For example, a potential underestimation of SB in single-item questionnaires was suggested [15, 104], whereas wider limits of agreement in questionnaires are present with multiple items [104]. Another factor contributing to validity of questionnaires may relate to the number of questions, and therefore detail of information, with more questions on SB potentially improving the criterion validity of the measurement tool. In contrast to this hypothesis, our analysis revealed no substantial differences between the criterion validity of the 1-item, 2-to-9-item and ≥ 10-item questionnaires. One possible explanation is that participants find it difficult to recall SB, with multiple-item questionnaires making it even more complicated to replicate detailed and domain-specific patterns of SB [31]. Furthermore, some behaviours are easier to remember because these are more habitual and restricted to certain periods during the day, e.g. TV viewing, computer use or sitting at work [15, 31, 86]. Finally, multiple-item questionnaires may over-report SB because subjects may report sedentary activities twice when using sub-scales (e.g. driving while listing to music). Although more questions may cover multiple domains and provide more detailed information, the complexity of these questionnaires may contribute to the negligible improvement in criterion validity of multiple-item questionnaires for total sedentary time. Nonetheless, exploring multiple domains of sitting may still seem relevant. For example, some domains are more strongly associated with poor health outcomes [12–14], whilst detailed information about domains may provide insight for intervention development.

### Reliability of subjective measures of SB

Despite the significant heterogeneity in validity of the various measures to assess SB, the reliability of the questionnaires and diaries or logs were moderate-to-good. Importantly, these conclusions are based on studies with a fair-to-good quality. A central question pertaining to the reliability of questionnaires is whether differences are present in reliability for weekdays versus weekend days or for workdays versus non-workdays, especially given the marked differences in (sedentary) behaviour that exist between these days [104]. Indeed, our study found that approximately 50% of included studies reported a ≥ 10% better reliability to assess SB during weekdays versus weekend days or during workdays versus non-workdays (Table 3). These observations support a previous review, which reported higher reliability for weekdays compared to weekend days [104]. Moreover, we found that reliability was better for specific behaviours, such as TV viewing, compared to a more general categories, such as ‘other leisure time activities’. An explanation for this finding is that more specific and regularly performed behaviours have a higher reliability [15].

### Choosing an appropriate measurement tool

Logs and diaries have a higher validity compared to the questionnaires, are less reliant on long-term recall and can more accurately capture sporadic and intermittent behaviours. Therefore, we recommend logs and dairies as self-reported measurement tools. However, important limitations such as time constrains, lack of resources and the potential to influence participants’ behaviour, make them less useful for large-scale observational studies and/or intervention studies. Within the spectrum of questionnaires, there is no obvious preference for a single questionnaire. In fact, the most appropriate tool seems to depend on the nature of the study, especially since this review showed large variety in both validity and questionnaire characteristics (i.e. type and context of SBs). Therefore, some studies will benefit from questionnaires focusing on specific domains of SB, whilst others will benefit from a reliable estimate of total sedentary time or distribution of SB. Furthermore, when performing an intervention study, measures will benefit from the ability to measure changes across time. Since this ability was not examined within this review, we cannot make specific recommendations related to this type of studies. Nonetheless, these characteristics should be taken into account when planning such studies. Ultimately, and when feasible, a combination of objective and subjective assessments is preferred to provide valid and reliable insight into SB.

## Conclusions

This review identified the widespread (and rapidly growing) use of a large range of self-reported measures of SB, which significantly differ in type, extensiveness, complexity and duration. Our results indicated that the criterion validity of subjective measures ranged between poor and excellent, whereas the quality of most studies was poor. The validity of the logs/diaries was significantly higher compared to the questionnaires, with little improvement in criterion validity of questionnaires when increasing items to assess SB. Therefore, when only self-report measures are feasible, logs and diaries are recommended to validly and reliably assess SB, but due to time constraints and resources related to logs and diaries, 1-item questionnaires may be preferred in large-scale studies when showing similar validity and reliability compared to longer questionnaires. Whenever feasible, the combination of objective and subjective assessments will provide the most valid and reliable method to assess SB.

## Supplementary information

**Additional file :1 Table S1.** Search strategy. **Table S2.** Assessing the quality of studies examining the criterion validity. **Table S3.** Assessing the quality of studies examining the reliability.

## Data Availability

Not applicable.

## Abbreviations

ICC: Intra Correlation Coefficients
MET: Metabolic Equivalent of Task
R: Correlation Coefficient Estimate
SB: Sedentary Behaviour
