# Supplementary material for: Validity and reliability of subjective methods to assess sedentary behaviour in adults: a systematic review and meta-analysis
Source: Int J Behav Nutr Phys Act. 2020 Jun 15;17:75. doi: 10.1186/s12966-020-00972-1 (PMC7294635; doi:10.1186/s12966-020-00972-1)
Supplement: Supplementary file 1 — Additional file :1 Table S1. Search strategy. Table S2. Assessing the quality of studies examining the criterion validity. Table S3. Assessing the quality of studies examining the reliability. [file 12966_2020_972_MOESM1_ESM.docx]

**ADDITIONAL FILES**

**Additional Table 1.** Search strategy

| **Database** | **Sedentary behaviour** | **Self-reported measures** | **Validity/reproducibility** |
| --- | --- | --- | --- |
| *MEDLINE* | Sedentary Lifestyle/  Sedentary.ti,ab,kw.  ((sitting or reclining) adj2 time) .ti,ab,kw.  Physical* inactive*.ti,ab,kw.  Screen time.ti,ab,kw.  ((Watch* or view*) adj (television or TV)) .ti,ab,kw.  ((television or TV) adj (viewing or watching or time)).ti,ab,kw.  (computer adj (time or "use")).ti,ab,kw. (play* adj (videogame* or "video game*" or "computer game*" or "electronic game*")).ti,ab,kw.  ((driving or commut*) adj2 time).ti,ab,kw. | "surveys and questionnaires"/ or health surveys/ or behavioral risk factor surveillance system/ or exp health status indicators/ or exp population surveillance/ or self report/  (survey or surveys or questionnaire or questionnaires or self-report* or diary or diaries or interview or interviews or subjective or recall or (activity and (log or logs))).ti,ab,kw. | exp "reproducibility of results"/  validation studies.pt.  exp "Sensitivity and Specificity"/  (validity or validation or validate or validated or reliable or reliability or reproducible or reproducibility or responsive or responsiveness or specificity).ti,ab,kw. |
| *EMBASE* | Sedentary Lifestyle/  Sedentary.ti,ab,kw.  ((sitting or reclining) adj2 time) .ti,ab,kw.  Physical* inactive*.ti,ab,kw.  Screen time.ti,ab,kw.  ((Watch* or view*) adj (television or TV)) .ti,ab,kw.  ((television or TV) adj (viewing or watching or time)).ti,ab,kw.  (computer adj (time or "use")).ti,ab,kw. (play* adj (videogame* or "video game*" or "computer game*" or "electronic game*")).ti,ab,kw.  ((driving or commut*) adj2 time).ti,ab,kw. | Exp questionnaire/ or health survey/ or health status indicator/ or self report  (survey or surveys or questionnaire or questionnaires or self-report* or diary or diaries or interview or interviews or subjective or recall or (activity and (log or logs))).ti,ab,kw. | Reproducibility/  "Sensitivity and Specificity"/  (validity or validation or validate or reliable or reliability or reproducible or reproducibility or responsive or responsiveness or specificity).ti,ab,kw.  Validation study/ or exp validity/ |
| *SPORTDiscus* | SEDENTARY Lifestyles OR SEDENTARY behavior  SITTING position  AB Sedentary OR TI sedentary  AB (((sitting or reclining) N2 (time)) OR TI (((sitting or reclining)  AB Physical* inactiv* OR TI Physical* inactiv*  AB screen time OR TI screen time  AB (((watch* OR view*) N (television OR TV)) OR TI (((watch* OR view*) N (television OR TV) )  AB (((television or TV) N (viewing or watching or time))) OR TI ( ((television or TV) N (viewing or watching or time)) )  AB ((computer N (time or “use”)) OR TI ((computer N (time or “use”))  AB ((play* N (videogame* or “video game*” or “computer game*” or “electronic game*”))) OR TI ( (play* N (videogame* or “video game*” or “computer game*” or “electronic game*”)) )  AB ((( driving or commut*) N2 time) ) OR TI ( (( driving or commut*) N2 time) ) | AB ((survey or surveys or questionnaire or questionnaires or self-report* or diary or diaries or interview or interviews or subjective or recall or (activity and (log or logs))) ) AND TI ( (survey or surveys or questionnaire or questionnaires or self-report* or diary or diaries or interview or interviews or subjective or recall or (activity and (log or logs)))) | AB ((validity or validation or validate or reliable or reliability or reproducibility or responsive or responsiveness or specificity) ) AND TI ( (validity or validation or validate or reliable or reliability or reproducibility or responsive or responsiveness or specificity)) |

**Additional Table 2.** Assessing the quality of studies examining the criterion validity.

|  | **Excellent** | **Good** | **Fair** | **Poor** |
| --- | --- | --- | --- | --- |
| **Design requirements** |  |  |  |  |
| Was the percentage of missing items given? | Percentage of missing items described | Percentage of missing items NOT described |  |  |
| Was there a description of how missing items were handled? | Described how missing items were handled | Not described but it can be deduced how missing items were handled | Not clear how missing items were handled |  |
| Was the sample size included in the analysis adequate? | Adequate sample size (≥100) | Good sample size (50-99) | Moderate sample size (30-49) | Small sample size (<30) |
| Can the criterion used or employed be considered as a reasonable ‘gold standard’? | Criterion used can be considered an adequate ‘gold standard’ (evidence provided) | No evidence provided, but assumable that the criterion used can be considered an adequate ‘gold standard’ | Unclear whether the criterion used can be considered an adequate ‘gold standard’ | Criterion used can NOT be considered an adequate ‘gold standard’ |
| Were there any important flaws in the design or methods of the study? | No other important methodological flaws in the design or execution of the study | Other minor methodological flaws in the design or execution of the study | Other important methodological flaws in the design or execution of the study | No other important methodological flaws in the design or execution of the study |
| **Statistical methods** |  |  |  |  |
| For continuous scores: Were correlations, or the area under the receiver operating curve calculated? | Correlations or AUC calculated |  |  | Correlations or AUC NOT calculated |
| For dichotomous scores: Were sensitivity and specificity determined? | Sensitivity and specificity calculated |  |  | Sensitivity and specificity NOT calculated |

Table adapted from Terwee CB, et al. Rating the methodological quality in systematic reviews of studies on measurement properties: a scoring system for the COSMIN checklist. Quality of Life Research. 2012.

**Additional Table 3.** Assessing the quality of studies examining the reliability.

|  | **Excellent** | **Good** | **Fair** | **Poor** |
| --- | --- | --- | --- | --- |
| **Design requirements** |  |  |  |  |
| Was the percentage of missing items given? | Percentage of missing items described | Percentage of missing items NOT described |  |  |
| Was there a description of how missing items were handled? | Described how missing items were handled | Not described but it can be deduced how missing items were handled | Not clear how missing items were handled |  |
| Was the sample size included in the analysis adequate? | Adequate sample size (≥100) | Good sample size (50-99) | Moderate sample size (30-49) | Small sample size (<30) |
| Were at least two measurements available? | At least two measurements |  | ? | Only one measurement |
| Were the administrations independent? | Independent measurements | Assumable that the measurements were independent | Doubtful whether the measurements were independent | measurements NOT independent |
| Was the time interval stated? | Time interval stated |  | Time interval NOT stated |  |
| Were patients stable in the interim period on the construct to be measured? | Patients were stable (evidence provided) | Assumable that patients were stable | Unclear if patients were stable | Patients were NOT stable |
| Was the time interval appropriate? | Time interval appropriate |  | Doubtful whether time interval was appropriate | Time interval NOT appropriate |
| Were the test conditions similar for both measurements? e.g. type of administration, environment, instructions | Test conditions were similar (evidence provided) | Assumable that test conditions were similar | Unclear if test conditions were similar | Test conditions were NOT similar |
| Were there any important flaws in the design or methods of the study? | No other important methodological flaws in the design or execution of the study |  | Other minor methodological flaws in the design or execution of the study | Other important methodological flaws in the design or execution of the study |
| **Statistical methods** |  |  |  |  |
| For continuous scores: Was an intraclass correlation coefficient (ICC) calculated? | ICC calculated and model or formula of the ICC is described | ICC calculated but model or formula of the ICC not described or not optimal.  Pearson or Spearman correlation coefficient calculated with evidence provided that no systematic change has occurred | Pearson or Spearman correlation coefficient calculated WITHOUT evidence provided that no systematic change has occurred or WITH evidence that systematic change has occurred | No ICC or Pearson or Spearman correlations calculated |
| For dichotomous/nominal/ordinal scores: Was kappa calculated? | Kappa calculated |  |  | Only percentage agreement calculated |
| For ordinal scores: Was a weighted kappa calculated? | Weighted Kappa calculated |  | Unweighted Kappa calculated | Only percentage agreement calculated |
| For ordinal scores: Was the weighting scheme described? e.g. linear, quadratic | Weighting scheme described | Weighting scheme NOT described |  |  |

Table adapted from Terwee CB, et al. Rating the methodological quality in systematic reviews of studies on measurement properties: a scoring system for the COSMIN checklist. Quality of Life Research. 2012.
